# Peripheral Blood Leukocyte Ratios as Novel Biomarkers in Brain Glioma: A Comprehensive Systematic Review and Meta‐Analysis

**DOI:** 10.1111/jcmm.70974

**Published:** 2026-01-08

**Authors:** Fatemeh Hasani, Kimia Jazi, Kimia Vakili, Armin Tafazolimoghadam, Mehrad Namazee, Mahdi Masrour, Mohammadreza Ghanbari Boroujeni, Hossein Gandomkar, Antonio L. Teixeira, Erfan Ghoodjani, Mohammad Samadian, Fatemeh Sayehmiri, Seyed Ali Mousavinejad

**Affiliations:** ^1^ Gastroenterology and Hepatology Research Center Golestan University of Medical Sciences Gorgan Iran; ^2^ Neuroscience Research Center Golestan University of Medical Sciences Gorgan Iran; ^3^ School of Medicine Shahid Beheshti University of Medical Sciences Tehran Iran; ^4^ School of Medicine Tehran University of Medical Sciences Tehran Iran; ^5^ School of Medicine Shiraz University of Medical Sciences Shiraz Iran; ^6^ Students Research Committee Shahrekord University of Medical Sciences Shahrekord Iran; ^7^ Department of Surgical Oncology Tehran University of Medical Medicine Tehran Iran; ^8^ Biggs Institute The University of Texas Health Science Center at San Antonio San Antonio Texas USA; ^9^ School of Medicine Isfahan University of Medical Sciences Isfahan Iran; ^10^ Skull Base Research Center, Loghman Hakim Hospital Shahid Beheshti University of Medical Sciences Tehran Iran

**Keywords:** biomarkers, glioma, leukocyte count, neutrophil‐to‐lymphocyte ratio, tumour

## Abstract

Inflammatory biomarkers, such as leukocyte ratios, have emerged as promising tools for diagnosing and prognosticating brain gliomas. This study systematically reviewed and analysed the diagnostic and prognostic relevance of peripheral blood leukocyte ratios in glioma. Following the PRISMA guidelines, we conducted a systematic review and meta‐analysis by searching PubMed, Web of Science, and Scopus for studies published in English. Eligible studies evaluated the sensitivity, specificity, and area under the curve (AUC) of inflammatory ratios, as well as their associations with survival outcomes. Quality was assessed using the Newcastle‐Ottawa Scale. A total of 29 assessments with 13,189 observations compared the neutrophil‐to‐lymphocyte ratio (NLR) between glioma patients and non‐glioma groups, yielding a pooled standardised mean difference (SMD) of 0.445 (95% CI: 0.280–0.609, *p* < 0.0001; *I* ^2^ = 85.1%). When compared to healthy individuals (10 assessments, 4444 observations), glioma patients exhibited a significantly elevated NLR (SMD: 0.797, 95% CI: 0.576–1.019, *p* < 0.0001; *I* ^2^ = 87.5%). Compared to meningioma (5 assessments, 3227 observations), glioma patients had a significantly higher NLR (SMD: 0.352, 95% CI: 0.280–0.424, *p* < 0.0001; *I* ^2^ = 24.7%). In comparisons with brain metastasis (4 assessments, 428 observations), the difference was not significant (SMD: −0.112, *p* = 0.3315; *I* ^2^ = 44.6%). The platelet‐to‐lymphocyte ratio (PLR) (25 assessments, 12,085 observations) showed no significant difference between glioma and non‐glioma groups (SMD: 0.1291, *p* = 0.0836; *I* ^2^ = 81.4%). Similarly, the derived NLR (dNLR) was significantly higher in glioma patients than in non‐glioma groups (SMD: 0.2421, *p* < 0.0001; *I* ^2^ = 49.9%). The lymphocyte‐to‐monocyte ratio (LMR) was significantly lower in glioma compared to meningioma (SMD: −0.2989, *p* < 0.0001; *I* ^2^ = 0.0%). MLR analysis showed high heterogeneity (*I* ^2^ = 99.5%) with non‐significant findings (*p* = 0.4476). These findings suggest NLR and dNLR as potential biomarkers for glioma diagnosis. Peripheral blood leukocyte ratios, particularly NLR, represent valuable biomarkers for glioma diagnosis and prognosis. Further research is warranted to enhance their precision and clinical utility.

## 1 Introduction

Brain and other central nervous system (CNS) tumours are among the most fatal forms of cancer, causing significant morbidity and mortality [1]. There were 83,029 deaths attributed to malignant brain tumours and other CNS malignancies in the US between 2014 and 2018, according to the most recent data from the Central Brain Tumour Registry of the United States (CBTRUS). This resulted in an average annual death toll of 16,606 and an average annual mortality rate of 4.43 per 100,000 people [2].

Glioma accounts for 81% of all CNS tumours, making it the most common primary brain tumour [3]. Within the spectrum of gliomas, glioblastoma (GBM) is the most aggressive and common subtype [4, 5]. GBM is also the most prevalent malignant brain tumour, constituting around 15% of all brain tumours and 50% of malignant brain tumours [2, 6, 7]. Even after combining radiation, immunotherapy, surgery, and tumour‐treating fields, the 5‐year survival rate for GBM is still poor [8, 9, 10, 11, 12]. In contrast to other cancers, glioma does not have specific or sensitive serum markers for tumour grading, treatment response monitoring, tumour identification, or prognostic assessment [13, 14, 15, 16].

While neuroimaging plays a role in preoperative evaluation and postoperative monitoring of treatment response and tumour recurrence, histopathological analysis has long been the gold standard for glioma diagnosis [17]. For that, there is the need for brain biopsy that can be costly, also carrying the risks of intracranial haemorrhage and neurological damage. Hence, there is the need for sensitive, cost‐effective, and specific biomarkers for diagnosing glioma, and also ideally grading and monitoring its recurrence and response to treatment [18, 19, 20].

The role of inflammatory responses in the pathogenesis of solid tumours has been shown in multiple studies. A close relationship has been established between inflammation and tumour oncogenesis, progression, treatment resistance, and prognosis [21, 22, 23, 24, 25]. Chronic inflammation within the brain microenvironment can contribute to glioma initiation, progression, and resistance to therapy [26]. Inflammation is also associated with changes in the circulating numbers of white blood cells (WBCs), neutrophils, lymphocytes, monocytes, and platelets. These blood‐based biomarkers are often cost‐effective and readily available for routine preoperative monitoring. Studies have now established the predictive role of circulating leukocyte ratios and their ratios in various solid tumours, including lung cancer [27], cervical cancer [28], penile cancer [29], colorectal cancer [30], thyroid carcinoma [31], bladder cancer [32], oesophageal squamous cell carcinoma [33], hilar cholangiocarcinoma [34], breast cancer [35], and others.

Studies investigating the diagnostic value of circulating inflammatory markers, such as neutrophil‐to‐lymphocyte ratio (NLR), platelet‐to‐lymphocyte ratio (PLR), etc. in glioma are few in comparison to other solid tumours [4, 36, 37]. Moreover, most research has focused on GBM patients' survival and recurrence. Comprehensive studies investigating the role of inflammatory markers and their ratios in glioma molecular typing, grading, and diagnosis are limited and have conflicting results. In this context, the objective of the current study was to compare the levels of a subset of major inflammatory markers in glioma patients and the control group. The utility of NLR, derived neutrophil‐to‐lymphocyte ratio (dNLR), PLR, lymphocyte‐to‐monocyte ratio (LMR), and prognostic nutritional index (PNI), and their combinations, in the diagnosis, grading, and molecular typing of glioma was also thoroughly examined.

## 2 Materials and Methods

This study is represented following the Preferred Reporting Items for Systematic Reviews and Meta‐Analyses (PRISMA) guidelines [38]. We have registered our systematic review and meta‐analysis protocol in PROSPERO under the registration number CRD42024504743.

### 2.1 Literature Search

On January 21, 2024, we ran a comprehensive search across several databases, including PubMed, Web of Science (ISI), and Scopus. Our search aimed to identify English publications without imposing any restrictions on the publication year. We utilized a combination of medical subject headings (MeSH) terms and free keywords, including “monocyte‐lymphocyte ratio”, “neutrophil‐lymphocyte ratio”, “platelet‐lymphocyte ratio” and “glioma”, along with their respective expansions, to search the databases. Further details on the search query are available in Table S1.

### 2.2 Selection Criteria

This study included original peer‐reviewed research focusing on the sensitivity, specificity, and area under the curve (AUC) values of the above‐mentioned ratios in glioma diagnosis. We also included studies that investigated how these ratios are related to prognosis using overall survival (OS), progression‐free survival (PFS), disease‐free survival (DFS), recurrence‐free survival (RFS), and event‐free survival (EFS). The studies were conducted prospectively or retrospectively, employing samples obtained from patients who had received a pathological diagnosis of glioma, as well as from healthy individuals serving as controls. When investigating diagnostic accuracy, it is imperative to compare the ratios of patients to those of appropriate controls, regardless of the duration of test assay time. Hence, studies with inappropriate controls were omitted [39].

Our predetermined eligibility criteria did not impose limitations on the healthcare settings where the research was conducted or the total number of participants for the included studies. Non‐English studies utilizing datasets or animal models, letters, comments, reviews, editorials, conference abstracts, case reports, and case series were deemed ineligible and consequently excluded from the study.

### 2.3 Study Selection and Data Extraction

After removing duplicates, F.H. and A.T. reviewed the remaining identified papers to assess their eligibility. This evaluation was based on the predefined inclusion and exclusion criteria. Once a list of studies meeting the eligibility criteria was compiled, both authors independently reviewed the full texts of these studies. During the review process, any conflicts that arose were effectively resolved through consensus‐building discussions between the authors. Two reviewers, F.H. and M.G., independently extracted data from the included studies into a dedicated electronic spreadsheet.

From each study, the following information was gathered when available: Authors, year of publication, type of specimen, sample size, inflammatory biomarkers, type of control population, difference in the inflammatory ratio levels of the patients relative to the control group, diagnostic sensitivity, specificity, and AUC with the corresponding 95% confidence interval (CI) and *p*‐values; mean, median, and hazard ratio (HR) for survival outcomes with the corresponding 95% CI and *p*‐values. Discrepancies were resolved through discussion and consensus between the authors responsible for data extraction.

### 2.4 Quality Assessment

The quality of the included studies was assessed using the Newcastle‐Ottawa Scale (NOS), a suitable tool for evaluating cohort and case–control studies [40]. Two reviewers, FH and MG, independently evaluated the quality of each study according to NOS criteria. Any discrepancies in the quality assessment were resolved through discussion or consultation with a third reviewer if necessary. The NOS evaluates studies based on three main categories of bias: selection, comparability, and outcome. Scores of 7 and above were considered ‘good’, scores between 2 and 6 were considered ‘fair’, and scores of 1 and below were considered ‘poor’ regarding quality assessment. The quality assessment of included articles is shown in Table S2.

### 2.5 Statistical Analysis

The statistical analyses and visualisations were performed using R version 4.2.2 (R Core Team, Vienna, Austria) with the “meta” package [41]. We employed the bias‐corrected Hedges' g standardised mean difference (SMD) to compare NLR, PLR, dNLR, LMR, and MLR indices between glioma patients and various control groups. This approach was selected as it accounts for differences in sample sizes across studies, enhancing the precision of effect size estimates [42, 43].

A meta‐analysis was conducted using a random‐effects model for all comparisons to account for the substantial heterogeneity among studies due to differences in study populations, methodologies, and measurement techniques. Subgroup analyses and univariate meta‐regression were performed to investigate potential sources of heterogeneity, including variations in study design and population demographics. The *I* ^2^ statistic quantified heterogeneity, with values exceeding 50% considered indicative of significant variability. *𝜏* ^2^ values provided an additional measure of between‐study variance.

When data were reported as medians and interquartile ranges or means with ranges, the means and standard deviations were estimated using established methods by Luo, Wan, and Shi [44, 45, 46, 47]. Statistical significance was defined as a *p*‐value below 0.05.

Forest plots were used to present pooled SMDs with their 95% confidence intervals, while bubble plots visualised relationships between potential moderating variables and SMDs. For each biomarker comparison, sensitivity and specificity analyses were performed to determine optimal cut‐off points, with corresponding area under the curve (AUC) values calculated to evaluate diagnostic performance.

We reported 95% prediction intervals (PI) for random‐effects models to reflect the range of effects expected in a new study. For each meta‐analysis with *k* studies, we computed:

$$\mathrm{PI}=\hat{\mu }\pm t_{1-\alpha /2,k-2}\sqrt{{\hat{\tau }}^{2}+\mathrm{SE}\;{\left(\hat{\mu }\right)}^{2}}$$

where $\hat{\mu }$ is the pooled SMD, SE($\hat{\mu }$) is its standard error, and $\hat{\tau }$ ^2^ is the between‐study variance. We used two‐sided *α* = 0.05. When *k* < 4, PIs were still presented but interpreted cautiously due to unstable degrees of freedom.

We evaluated potential small‐study effects using funnel plots and Egger's regression test. Publication bias analyses were performed in Stata 17 (metabias, metafunnel), with *p* < 0.05 considered suggestive of asymmetry for Egger's test and trim‐and‐fill planned only when asymmetry was indicated.

## 3 Results

### 3.1 Characteristics of Included Studies

**FIGURE 1:**
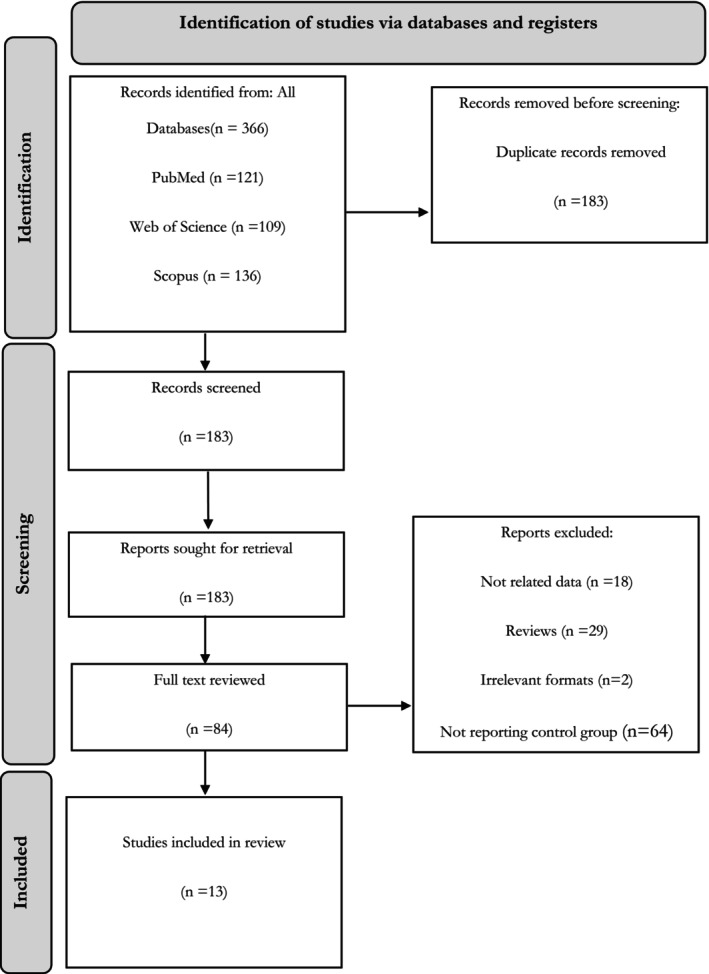
The PRISMA flow diagram representing the selection process of eligible studies.

The preliminary search yielded 366 records: 121 from PubMed, 136 from SCOPUS, and 109 from Web of Science. Following the elimination of 183 duplicates, 183 articles remained. Following the title and abstract screening of the remaining articles, the 84 titles were deemed suitable for full‐text screening. Seventy articles were excluded for multiple reasons (Figure 1). Finally, 13 studies were included in the systematic review. The characteristics of included studies in case of diagnosis are provided in Table S3.

### 3.2 NLR

**FIGURE 2:**
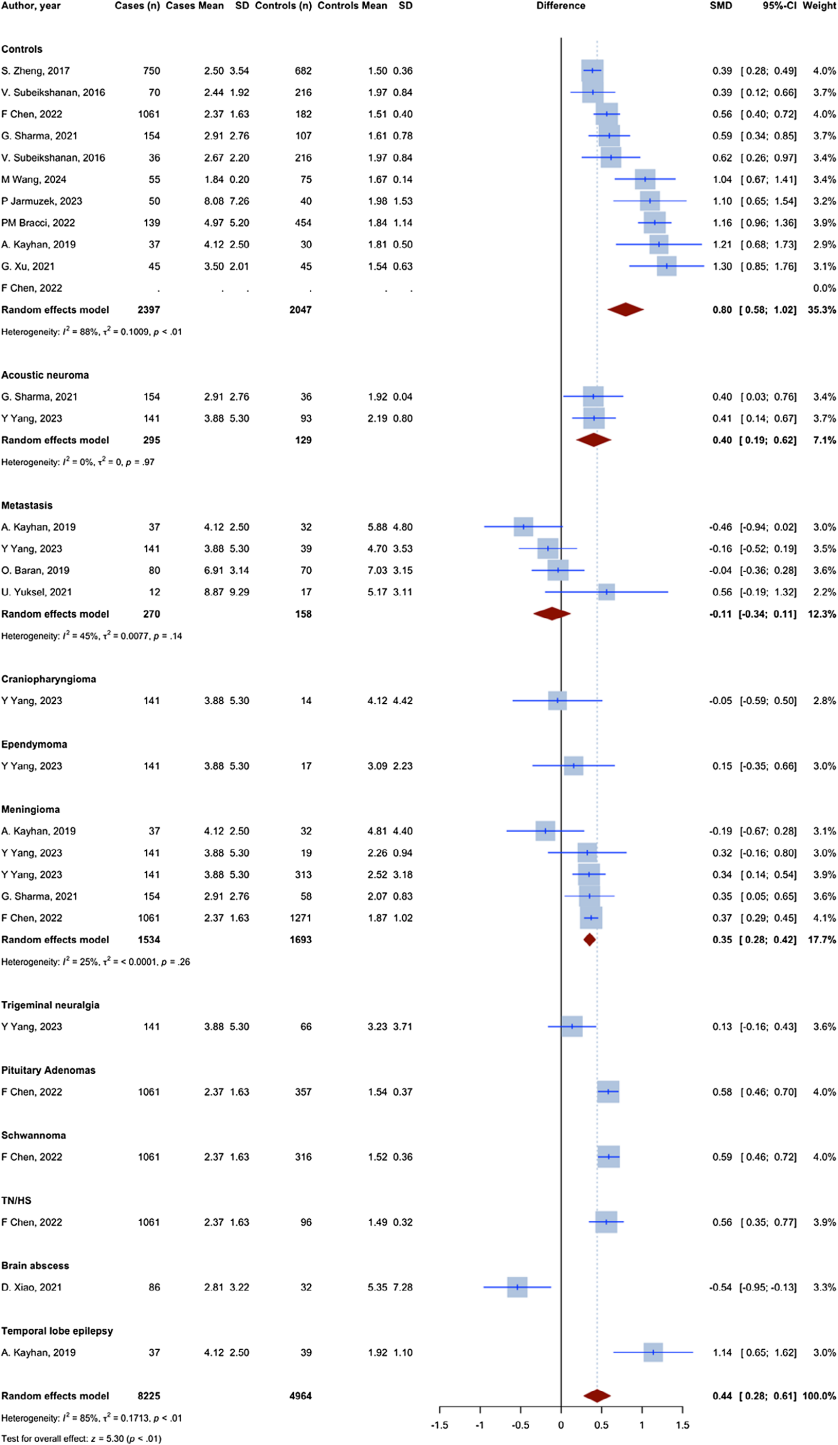
Meta‐analysis of calculated SMDs for NLR index.

Overall, 29 assessments with 13,189 observations compared the NLR of glioma patients with non‐glioma groups. The meta‐analysis yielded a pooled SMD of 0.445 (95% CI: 0.280; 0.609, 95% PI: −0.204 to 0.848; *p* < 0.0001; *I* ^2^ = 85.1%) (Figure 2).

10 assessments with 4444 observations compared the NLR of glioma patients with a group of individuals without any medical conditions. All assessments demonstrated a higher NLR index in patients with glioma, as indicated by a positive SMD. The meta‐analysis yielded a pooled SMD of 0.797 (95% CI: 0.578; 1.019, *p* < 0.0001; *I* ^2^ = 87.5%), indicating a significant increase in the NLR index in glioma patients compared to healthy controls.

Five assessments with 3227 observations have compared the NLR in glioma patients with meningioma patients. Four assessments showed a higher NLR index in glioma patients, as evidenced by a positive SMD, and one showed a lower NLR, as evidenced by a negative SMD. The meta‐analysis resulted in a combined SMD of 0.352 (95% CI: 0.280–0.424, *p* < 0.0001; *I* ^2^ = 24.7%), indicating a statistically significant increase in the NLR index in glioma compared to meningioma.

Four assessments with 428 observations have compared the NLR in glioma patients with brain metastasis patients. Three assessments showed a lower NLR index in glioma patients, as evidenced by a negative SMD, and one showed a higher NLR, as evidenced by a positive SMD. The meta‐analysis resulted in a combined SMD of −0.112 (95% CI: −0.339; 0.114, *p* = 0.332; *I* ^2^ = 44.6%).

In the comparison between glioma cases and all other controls, the optimal cut‐off point for the NLR was 2.35, yielding a sensitivity of 0.62 and a specificity of 0.72, corresponding to an AUC of 0.68. In comparing cases to healthy controls, the optimal cut‐off point for the NLR was determined to be 2.46, exhibiting a sensitivity of 0.61, a specificity of 0.85, and an AUC of 0.75. The cut‐off for comparison to metastasis was 4.56, with a sensitivity of 0.38, a specificity of 0.73, and an AUC of 0.56. In meningioma comparison, a cut‐off of 2.91 exhibited a sensitivity of 0.49, a specificity of 0.75, and an AUC of 0.63. The threshold for acoustic neuroma compared to glioma cases was 3.52, resulting in a sensitivity of 0.56, a specificity of 0.95, and an AUC of 0.74 (Table 1).

**TABLE 1:** The optimal cut‐off points and corresponding sensitivity, specificity, and AUC values for indices when comparing glioma cases to different types of controls.

	NLR				PLR				dNLR				LMR				MLR			
	Optimal cut‐off point	Sensitivity	Specificity	AUC	Optimal cut‐off point	Sensitivity	Specificity	AUC	Optimal cut‐off point	Sensitivity	Specificity	AUC	Optimal cut‐off point	Sensitivity	Specificity	AUC	Optimal cut‐off point	Sensitivity	Specificity	AUC
Vs. all controls	2.35	0.62	0.72	0.68	160	0.35	0.8	0.57	1.83	0.4	0.83	0.62	6.5	0.76	0.35	0.55				
Vs. healthy controls	2.46	0.61	0.85	0.75	143.36	0.44	0.8	0.61	1.78	0.48	0.89	0.67	6.9	0.82	0.27	0.5	0.21	0.52	0.85	0.65
Vs. metastasis	4.56	0.38	0.73	0.56	116.21	0.36	0.71	0.52					2.73	0.76	0.37	0.55				
Vs. meningioma	2.91	0.49	0.75	0.63	176.46	0.26	0.83	0.53	1.92	0.36	0.75	0.58	6.26	0.72	0.42	0.58				
Vs. acoustic neuroma	3.52	0.56	0.95	0.74	206.19	0.37	0.78	0.57	1.52	0.47	0.96	0.7								

### 3.3 PLR

**FIGURE 3:**
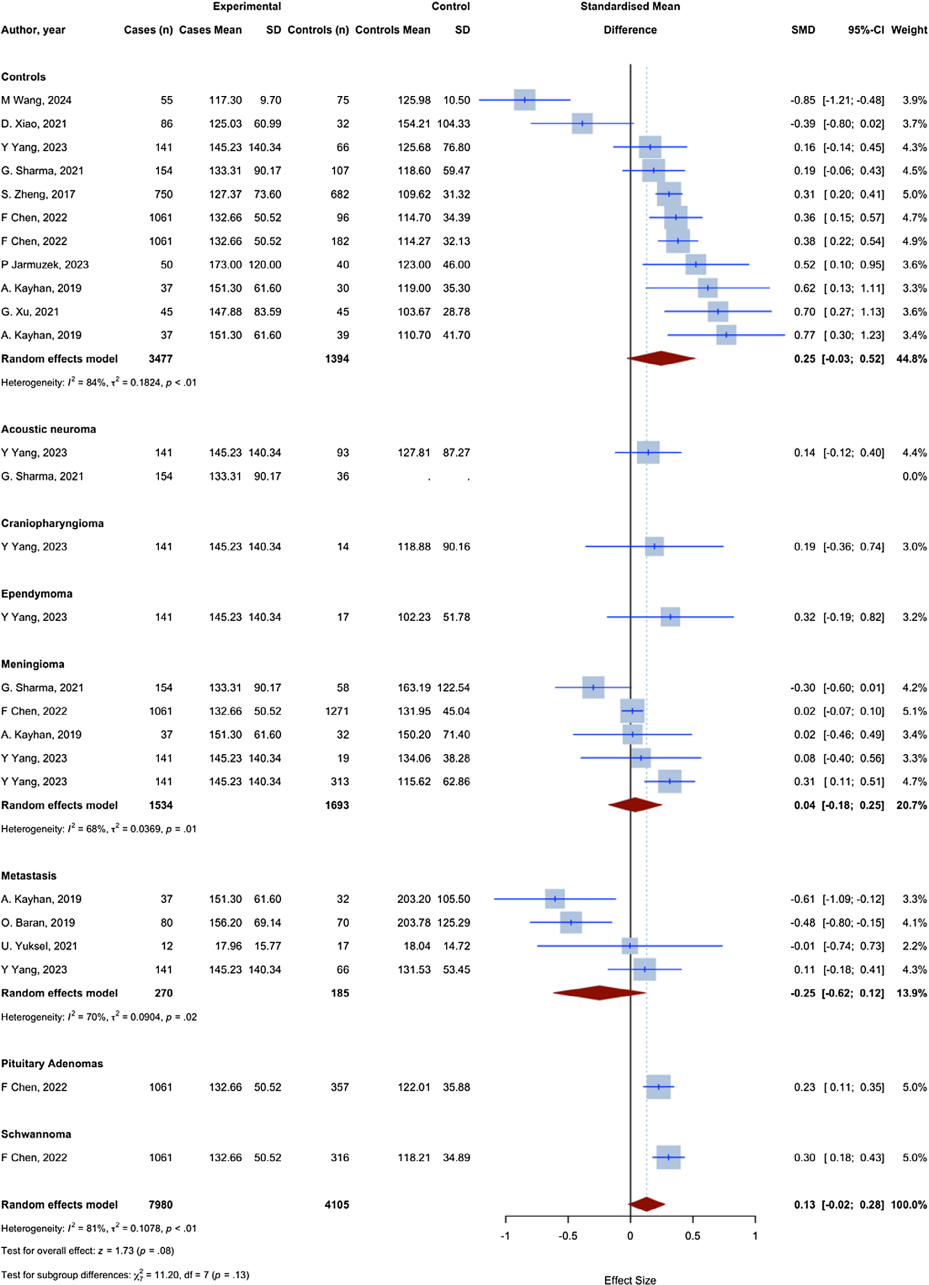
Meta‐analysis of calculated SMDs for PLR index.

Overall, 25 assessments with 12,085 observations compared the PLR of glioma patients with non‐glioma groups. The meta‐analysis yielded a pooled SMD of 0.129 (95% CI: −0.017; 0.275, 95% PI: −0.281 to 0.406; *p* = 0.084; *I* ^2^ = 81.4%) (Figure 3).

Eleven assessments with 4871 observations compared the PLR of glioma patients with a group of individuals without any medical conditions. Two assessments showed a lower PLR index in glioma patients, as evidenced by a negative SMD, and 9 showed a higher PLR, as evidenced by a positive SMD. The meta‐analysis yielded a pooled SMD of 0.245 (95% CI: −0.027; 0.518, *p* = 0.0773; *I* ^2^ = 83.9%).

Five assessments with 3227 observations have compared the PLR in glioma patients with meningioma patients. Four assessments showed a higher PLR index in glioma patients, as evidenced by a positive SMD, and one showed a lower PLR, as evidenced by a negative SMD. The meta‐analysis resulted in a combined SMD of 0.038 (95% CI: −0.176; 0.251, *p* = 0.7287; *I* ^2^ = 67.8%).

Four assessments with 455 observations have compared the PLR in glioma patients with brain metastasis patients. Three assessments showed a lower PLR index in glioma patients, as evidenced by a negative SMD, and one showed a higher PLR, as evidenced by a positive SMD. The meta‐analysis resulted in a combined SMD of −0.249 (95% CI: −0.618; 0.1194, *p* = 0.1850; *I* ^2^ = 70.4%).

In the comparison between glioma cases and all other controls, the optimal cut‐off point for the PLR was 160, yielding a sensitivity of 0.35 and a specificity of 0.8, which corresponds to an AUC of 0.57. In the comparison of cases to healthy controls, the optimal cut‐off point for the PLR was determined to be 143.36, exhibiting a sensitivity of 0.44, a specificity of 0.8, and an AUC of 0.61. The cut‐off for comparison to metastasis was 116.21, with a sensitivity of 0.36, a specificity of 0.71, and an AUC of 0.52. In meningioma comparison, a cut‐off of 176.46 exhibited a sensitivity of 0.26, a specificity of 0.83, and an AUC of 0.53. The threshold for acoustic neuroma in comparison to glioma cases was 206.19, resulting in a sensitivity of 0.37, a specificity of 0.78, and an AUC of 0.57.

### 3.4 dNLR

**FIGURE 4:**
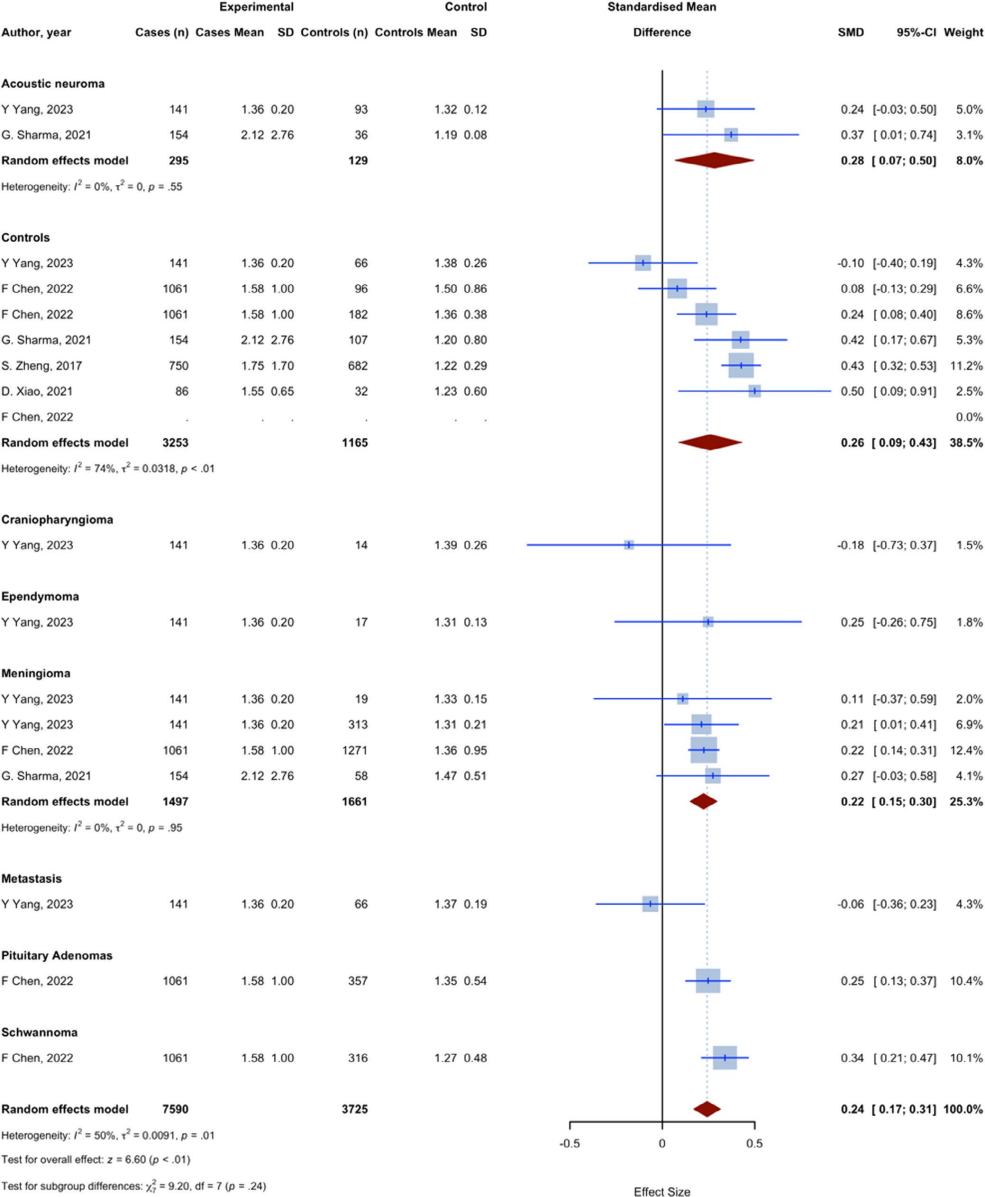
Meta‐analysis of calculated SMDs for dNLR index.

Overall, 17 assessments with 11,315 observations compared the dNLR (offers a reasonable estimate of the NLR) of glioma patients with non‐glioma groups. The meta‐analysis yielded a pooled SMD of 0.242 (95% CI: 0.170; 0.314, 95% PI −0.083 to 0.443; *p* < 0.0001; *I* ^2^ = 49.9%) (Figure 4).

Six assessments with 4418 observations compared the dNLR of glioma patients with a group of individuals without any medical conditions. One assessment showed a lower dNLR index in glioma patients, as evidenced by a negative SMD, and 5 showed a higher dNLR, as evidenced by a positive SMD. The meta‐analysis yielded a pooled SMD of 0.260 (95% CI: 0.088–0.431, *p* = 0.0030; *I* ^2^ = 74.3%), indicating a statistically significant increase in the dNLR index in glioma compared to healthy controls.

Four assessments with 3158 observations compared the dNLR in glioma patients with meningioma patients. All assessments showed a higher dNLR index in glioma patients, as evidenced by a positive SMD. The meta‐analysis resulted in a combined SMD of 0.223 (95% CI: 0.150; 0.295, *p* < 0.0001; *I* ^2^ = 0.0%), indicating a statistically significant increase in the dNLR index in glioma compared to meningioma.

In the comparison between glioma and all other controls, the optimal cut‐off point for the dNLR was 1.83, yielding a sensitivity of 0.4 and a specificity of 0.83, which corresponds to an AUC of 0.62. In the comparison of cases to healthy controls, the optimal cut‐off point for the dNLR was determined to be 1.78, exhibiting a sensitivity of 0.48, a specificity of 0.89, and an AUC of 0.67. The cut‐off for comparison to metastasis was 1.92, with a sensitivity of 0.36, a specificity of 0.75, and an AUC of 0.58. In the meningioma comparison, a cut‐off of 1.92 exhibited a sensitivity of 0.36, a specificity of 0.75, and an AUC of 0.58. The threshold for acoustic neuroma in comparison to glioma cases was 1.52, resulting in a sensitivity of 0.47, a specificity of 0.96, and an AUC of 0.7.

### 3.5 LMR

**FIGURE 5:**
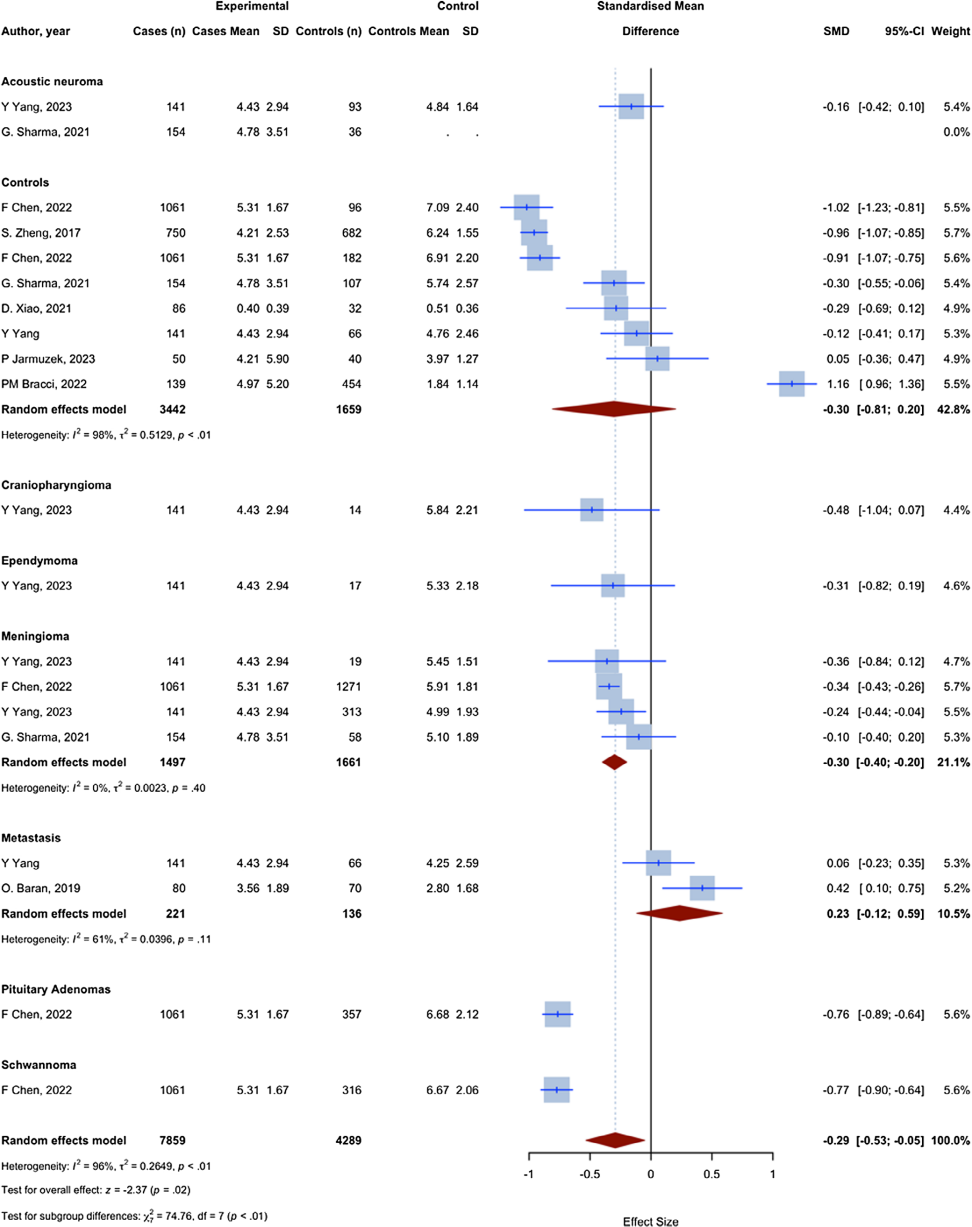
Meta‐analysis of calculated SMDs for LMR index.

Overall, 19 assessments with 12,148observations compared the LMR of glioma patients with non‐glioma groups. The meta‐analysis yielded a pooled SMD of −0.293 (95% CI: −0.534; −0.051, 95% PI −1.036 to 0.622; *p* = 0.0176; *I* ^2^ = 96.4%) (Figure 5).

Eight assessments with 5101 observations compared the LMR of glioma patients with a group of individuals without any medical conditions. Two assessments showed a higher LMR index in glioma patients, as evidenced by a positive SMD, and 6 showed a lower LMR, as evidenced by a negative SMD. The meta‐analysis yielded a pooled SMD of −0.303 (95% CI: −0.809; 0.202, *p* = 0.240; *I* ^2^ = 98.2%).

Four assessments with 3158 observations compared the LMR in glioma patients with meningioma patients. All assessments showed a lower LMR index in glioma patients, as evidenced by a negative SMD. The meta‐analysis resulted in a combined SMD of −0.299 (95% CI: −0.399; −0.199, *p* < 0.0001; *I* ^2^ = 0.0%), indicating a statistically significant decrease in the LMR index in glioma compared to meningioma.

In the comparison between glioma cases and all other controls, the optimal cut‐off point for the LMR was 0.76, yielding a sensitivity of 0.35 and a specificity of 0.55, which corresponds to an AUC of 0.55. In the comparison of cases to healthy controls, the optimal cut‐off point for the LMR was determined to be 0.82, exhibiting a sensitivity of 0.27, a specificity of 0.5, and an AUC of 0.5. The cut‐off for comparison to metastasis was 0.76, with a sensitivity of 0.37, a specificity of 0.55, and an AUC of 0.55. In the meningioma comparison, a cut‐off of 0.72 exhibited a sensitivity of 0.42, a specificity of 0.58, and an AUC of 0.58.

### 3.6 MLR

**FIGURE 6:**
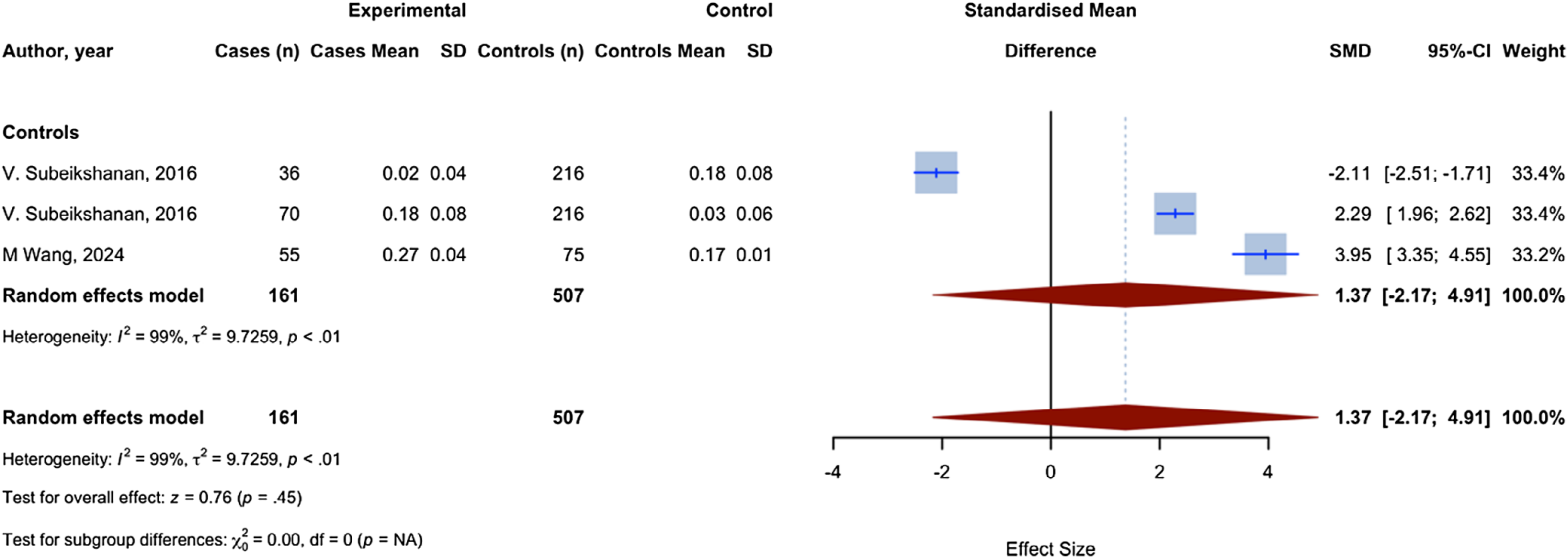
Meta‐analysis of calculated SMDs for MLR index.

Three assessments with 668 observations compared the MLR of glioma patients with a group of individuals without any health conditions. Two assessments showed a higher MLR index in glioma patients, as evidenced by a positive SMD, and one showed a lower MLR, as evidenced by a negative SMD. The meta‐analysis yielded a pooled SMD of 1.371 (95% CI: −2.168; 4.910, 95% PI −13.033 to 15.431 (*very imprecise given k = 3 and substantial heterogeneity*); *p* = 0.4476; *I* ^2^ = 99.5%) (Figure 6). In the comparison between glioma cases and healthy controls, the optimal cut‐off point for the MLR was 0.21, yielding a sensitivity of 0.52 and a specificity of 0.85, which corresponds to an AUC of 0.65.

### 3.7 Publication Bias

**FIGURE 7:**
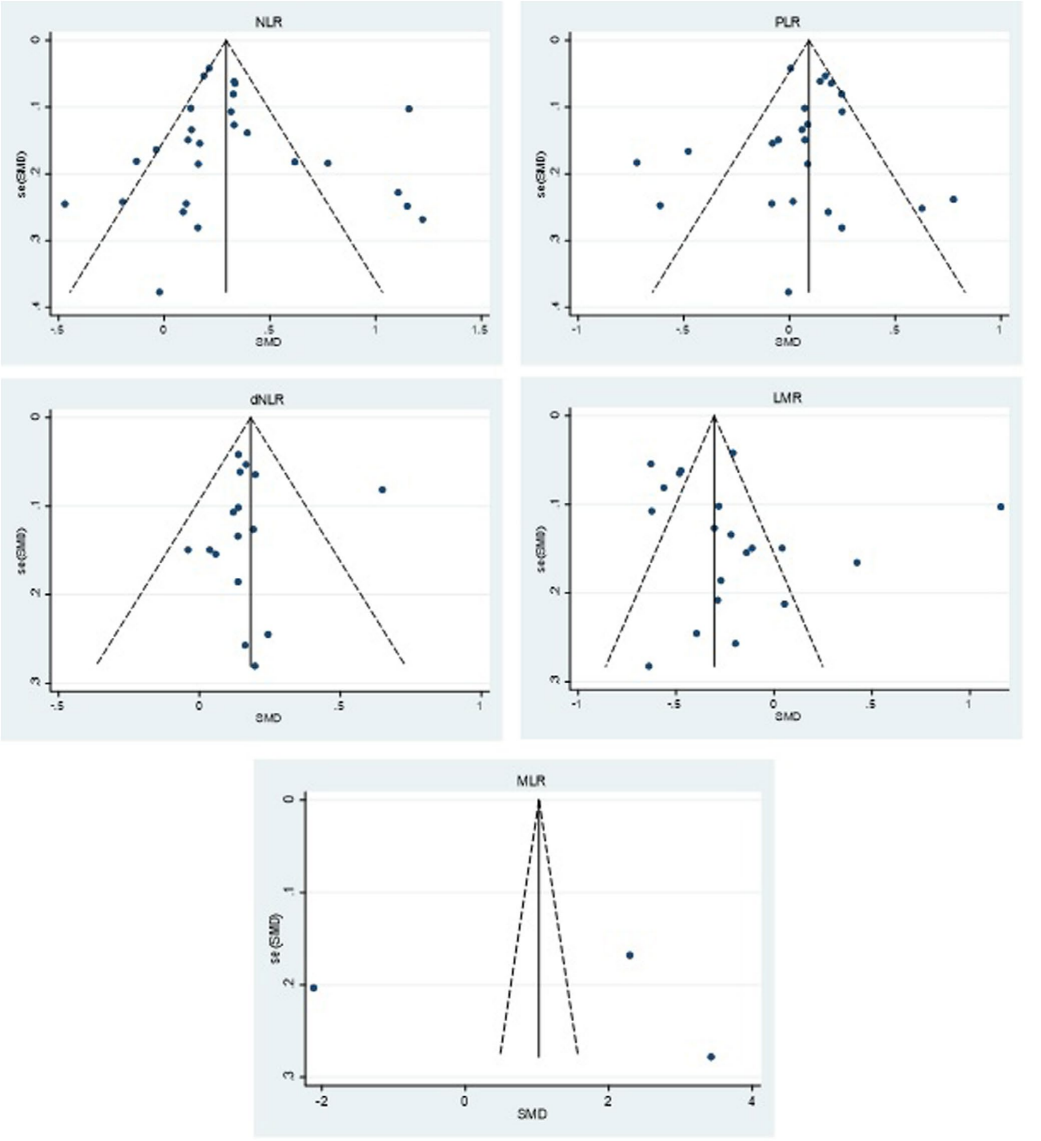
Funnel plots. Effect size (SMD, Hedges' g) on the x‐axis versus standard error on the y‐axis (inverted scale); the vertical line marks the pooled random‐effects estimate and dashed lines indicate pseudo 95% confidence limits. Visual inspection shows no marked asymmetry in either of the plots.

Funnel plots for NLR (*k* = 27), PLR (*k* = 23), dNLR (*k* = 16), and LMR (*k* = 20) did not show marked asymmetry, and Egger's test of the intercept was non‐significant in each: NLR *p* = 0.552, PLR *p* = 0.484, dNLR *p* = 0.880, LMR *p* = 0.302 (Figure 7A–D). For MLR (*k* = 3), Egger's test was *p* = 0.929 but was considered underpowered at this sample size; we therefore only considered a qualitative evaluation of funnel plots (Figure 7E).

## 4 Discussion

According to recent studies, inflammation is a hallmark of cancer [48]. The infiltrating leukocytes are crucial for tumour migration and dissemination, with a marked increase of inflammatory markers circulating in the blood [49, 50, 51]. Circulating monocytes or macrophages, or existing as brain‐resident microglia, represent 30%–50% of immune cells infiltrating glioblastoma (GBM). IL‐8, IL‐6, and CXCL1 are factors involved in neutrophil recruitment, and neutrophils contribute to tumour progression [52, 53]. They secrete VEGF, MMP‐9, and arginase‐1, resulting in angiogenesis and suppression of the immune system [54, 55]. Several treatment strategies against inflammation have been shown to be promising in glioma treatment. Tumour‐associated neutrophils (TANs) have also been targeted by using dapsone to reduce levels of VEGF and IL‐8‐induced neutrophilia [56, 57], or by exploiting them as delivery vehicles for liposomal paclitaxel in inflammatory glioma microenvironments [58]. Adjuvant treatment with non‐steroidal anti‐inflammatory drugs (NSAIDs) such as aspirin, celecoxib, and diclofenac also facilitates glioma control by suppressing COX‐2, NF‐κB, and STAT3 activation [59, 60] also points to inflammation as an important target in the development of novel, multi‐modal therapeutic approaches to glioma.

The current systematic review attempted to clarify the clinical relevance of certain inflammatory biomarkers in gliomas through meta‐analysis. Our results showed that, compared to healthy controls, glioma patients had significantly higher levels of NLR, dNLR, and PLR.

The pooled SMD for NLR index was significantly higher in glioma patients than in healthy controls, with an SMD of 0.7972 (95% CI: 0.5757–1.0186). Comparing glioma patients with all controls and healthy groups also revealed optimal cut‐off points of 2.35 (sensitivity: 0.62; specificity: 0.72; AUC: 0.68) and 2.46, respectively. In line with these findings, Gandhi et al. demonstrated that when comparing WHO grade II gliomas and healthy controls, an NLR > 2.75 was associated with diffuse glioma diagnosis [61]. Auezova et al. also reported a remarkable increase in NLR of grade I‐III and GBM patients with a cutoff point of 4, showing a poor prognosis [62]. A systematic review by Gomes et al. approved the association of high NLR values and high‐grade gliomas [63]. Moreover, Wilson and colleagues showed a considerably lower neutrophil count in low‐grade glioma paediatric patients compared to high‐grade, with a cut‐off of 3.36 as the predictor of death at 2 years post‐operation [64]. The prognostic significance of elevated NLR and unfavourable overall survival (HR: 1.43, 95% CI: 1.27–1.62) was highlighted in a recent meta‐analysis of 16 cohorts involving 2275 patients [65]. A study on 3101 cases compared to 182 healthy subjects showed that glioma patients had the highest preoperative NLR ratios with an AUC of 0.8099 (95% CI: 0.7823–0.8374). For GBM patients, the AUC value was 0.9585 (95% CI: 0.9467–0.9703) [66]. NLR had high sensitivity and specificity diagnostic value among all grades of glioma. In contrast to lymphocytes, neutrophil count was significantly increased in high‐grade gliomas, confirming the significant preoperative value for disease progression and higher grades [67]. In the groups with lower NLR levels, Isocitrate dehydrogenase (IDH) mutation was more frequent [68, 69], suggesting the possible immunosuppression induced by IDH mutation. Another study also concluded that tumour‐infiltrating lymphocytes were higher among IDH mutant glioma patients than wild‐type glioma [70]. This could explain the correlation between better survival and lower NLR. Moreover, we suggest that NLR accuracy and cutoff could be different among patients with neutrophil or lymphocyte subtypes.

Comparing meningioma and glioma patients, we found that NLR also increased with a pooled SMD of 0.3519 (95% CI: 0.2798–0.4240). Our findings determined that NLR > 2.91 would be an optimal cut‐off point. In contrast, a recent meta‐analysis of 13 studies assessing the prognostic and diagnostic value of NLR in meningioma reported that there was no difference in NLR among patients with glioma and meningioma (SMD: −0.19, 95% CI: −0.47–0.10, *p* = 0.20). This discrepancy could be due to different cut‐off values or other factors affecting NLR such as glioma grading, surgery, and chemotherapy. We did not find significant differences among patients with brain metastasis and glioma (SMD: −0.1122, 95% CI: −0.3388–0.1143, *p* = 0.3315). Moreover, NLR > 4.56 had the sensitivity, specificity, and AUC of 0.38, 0.73, and 0.56, respectively.

Our results also supported the previous conclusions that dNLR, LMR, and PLR might be useful biomarkers. In a study by Yang et al., the NLR in the brain metastasis group was lower compared to healthy controls, while NLR and NLR + dNLR showed the highest diagnostic value for glioma with AUC of 0.7490 (0.6482–0.8498) and 0.7481 (0.6457–0.8505), respectively [71]. Comparing PLR and dNLR values in glioma patients with healthy controls, this meta‐analysis showed a significant increase in both values with SMD of 0.2454 (95% CI: −0.0269; 0.5178) and 0.2595 (95% CI: 0.0880; 0.4310), respectively. In terms of LMR, findings yielded a pooled SMD of −0.3033 (95% CI: −0.8089; 0.2023, *p* = 0.2397) for LMR comparing glioma patients and healthy controls; however, when compared to the non‐glioma group, a pooled SMD of −0.293 (95% CI: −0.534; −0.051) suggested a possible significant reverse association of LMR levels and glioma diagnosis. In line with our results, although PLR was higher in preoperative glioma patients, it did not show statistical significance [66]. Han et al. also demonstrated that NLR was a stronger prognostic factor than PLR, despite their relation (*R* = 0.509) [37]. In contrast, studies also reported that the grade of glioma has been associated with PLR and dNLR increase and LMR decrease [64, 72]. Similarly, in contrast to the findings of the current analysis, LMR has shown the highest accuracy in the diagnosis and prognosis of glioma in combination with NLR [4] A retrospective study on 64 glioma patients revealed that pre‐treatment NLR (> 4.7) and LMR (> 0.35) were not only associated with poorer overall survival but also larger tumour diameter [73]. Zheng et al. investigated the diagnostic significance of NLR, dNLR, PLR, and LMR, and their combinations for glioma in different grades in a cohort study consisting of grade one to four glioma [74], as well as healthy controls, acoustic neuroma, meningioma, and non‐lesional epilepsy. The reported diagnostic AUCs were 0.722 (0.697–0.747) for NLR, 0.696 (0.670–0.722) for dNLR, 0.576 (0.549–0.604) for PLR, and 0.760 (0.738–0.783) for LMR [4]. Although the results on PLR analysis were not remarkable, this meta‐analysis also resulted in a statistically significant rise in the dNLR and LMR in glioma compared to meningioma patients. Disparities in the role of LMR and PLR across studies could be attributed to different tumour grades, tumour types, variations in biomarker measurement timing, and patients' characteristics.

According to Zheng et al., the best diagnostic value was for NLR + LMR and dNLR+LMR, with AUCs of 0.777 and 0.778, respectively [4]. Comparing grade I–III gliomas and GBM patients, NLR + LMR also demonstrated the greatest diagnostic accuracy. Authors concluded that the NLR + LMR combination could be a cost‐effective noninvasive biomarker with high sensitivity and specificity for glioma diagnosis in addition to the differential diagnosis of glioma from acoustic neuroma and meningioma, as well as GBM [4]. Additionally, Chen et al. determined that a combination of NLR + dNLR also had a significantly maximum diagnostic value (AUC: 0.8070; 0.7849–0.8291) for glioma [66]. The combination indices had shown the most valuable diagnostic and prognostic accuracy. Nevertheless, few studies have assessed the levels in glioma, and only dual combinations have been assessed. There is a lack of evidence on differentiating glioma from other neurological conditions, in addition to preoperative tumour grade to clarify the surgical outcome.

This is the first study providing a broad comparative meta‐analysis of inflammatory indices (NLR, dNLR, PLR, and LMR) across glioma patients with different gradings and control groups of healthy individuals, meningioma, and patients with brain metastasis, reporting the diagnostic and prognostic validity of markers along with cut‐off points. This meta‐analysis shows how leukocyte‐based biomarkers, including NLR and dNLR, can be used to diagnose and predict outcomes, particularly in glioma, by combining data from various tumour types, control groups, and inflammatory ratios. Unlike prior studies that primarily focused on survival or high‐grade glioma alone, our analysis provides a detailed comparative evaluation across glioma subtypes and relevant differential diagnoses, including meningioma and brain metastases. Importantly, we also identify the best cut‐off values for NLR, dNLR, and LMR when compared to different control groups, making them more useful in real‐life medical situations. This work offers clinicians a cost‐effective, non‐invasive adjunct to conventional imaging and histopathological approaches, particularly in preoperative triage and tumour grading. Additionally, by combining data from more than 13,000 cases, this study makes these markers more relevant for diagnosing gliomas and helps support their use in various diagnostic methods.

The growing interest in blood‐based biomarkers for glioma management provides an important contextual framework for interpreting the significance of peripheral leukocyte ratios. While markers such as the neutrophil‐to‐lymphocyte ratio, lymphocyte‐to‐monocyte ratio, and related indices offer easily accessible and inexpensive indicators of systemic immune dysregulation, recent literature underscores that these parameters represent only one component of a broader spectrum of circulating biomarkers. As outlined in the review by Goutnik and Lucke‐Wold [75], serum‐derived markers—including inflammatory proteins, immune mediators, and multiplex microRNA panels—have demonstrated potential to reflect tumour activity, treatment‐related changes, and the balance between recurrence and radiation‐induced injury. Their analysis emphasizes that although single analytes often lack sufficient sensitivity or specificity, integrating leukocyte‐based inflammatory measures with targeted serum biomarker panels may generate a more accurate and clinically meaningful picture of disease biology. This approach positions leukocyte ratios not as isolated predictors but as complementary indicators within a multi‐layered diagnostic framework that captures both systemic immune responses and tumour‐associated molecular signals.

Parallel to advancements in biomarker discovery, therapeutic strategies for glioma are increasingly oriented toward selective chemotherapy and molecularly guided treatment algorithms. Contemporary research is shifting from broadly cytotoxic approaches toward therapies tailored to tumour‐specific vulnerabilities, including metabolic dependencies, receptor‐mediated pathways, and the immunosuppressive microenvironment characteristic of high‐grade gliomas [76]. This paradigm highlights the need for reliable stratification tools capable of identifying patients most likely to benefit from such targeted interventions. Integrating peripheral leukocyte ratios with serum biomarker data offers a promising avenue in this regard. Elevated inflammatory ratios may reflect an immune‐suppressive milieu or aggressive tumour behaviour, while concurrent serum profiling could reveal actionable molecular features or resistance patterns. Combining these dimensions could enable clinicians to classify patients into biologically distinct subgroups, thereby informing whether a targeted agent, combination therapy, or intensified cytotoxic regimen is most appropriate.

Taken together, the convergence of serum biomarker utilisation and selective chemotherapy development suggests a forward‐looking clinical model in which peripheral leukocyte ratios function as a first‐tier screening measure that signals the need for deeper molecular characterisation. Subsequent analysis of serum‐based markers—especially multiplex panels capturing cytokines, chemokines, and tumour‐associated microRNAs—could refine therapeutic decision‐making and support personalised treatment planning. For glioma, where tissue acquisition is often limited and disease heterogeneity is substantial, such a blood‐based stratification pathway may help overcome current diagnostic constraints and accelerate translation of targeted therapies into practice. Future studies should therefore examine leukocyte ratios alongside comprehensive serum biomarker profiling and evaluate whether these combined indicators can predict responsiveness to emerging selective chemotherapy regimens. Incorporating these elements into prospective clinical designs will be essential for determining how blood‐based biomarkers can move beyond prognostic association to play a direct role in therapeutic selection.

This is the first study providing a broad comparative meta‐analysis of inflammatory indices (NLR, dNLR, PLR, and LMR) across glioma patients with different gradings and control groups of healthy individuals, meningioma, and patients with brain metastasis, reporting the diagnostic and prognostic validity of markers along with cut‐off points. This meta‐analysis shows how leukocyte‐based biomarkers, including NLR and dNLR, can be used to diagnose and predict outcomes, particularly in glioma, by combining data from various tumour types, control groups, and inflammatory ratios. Unlike prior studies that primarily focused on survival or high‐grade glioma alone, our analysis provides a detailed comparative evaluation across glioma subtypes and relevant differential diagnoses, including meningioma and brain metastases. Importantly, we also identify the best cut‐off values for NLR, dNLR, and LMR when compared to different control groups, making them more useful in real‐life medical situations. This work offers clinicians a cost‐effective, non‐invasive adjunct to conventional imaging and histopathological approaches, particularly in preoperative triage and tumour grading. Additionally, by combining data from more than 13,000 cases, this study makes these markers more relevant for diagnosing gliomas and helps support their use in various diagnostic methods.

There are limitations in this study that must be acknowledged. First, most of the studies were retrospectively designed. Second, most of the included studies were from single regions (e.g., China, USA, Europe). Future investigations should include other populations according to the important role of race or ethnicity in glioma. Third, in this study, we couldn't compare the inflammatory indices in pre‐ or post‐operative, recurrent or non‐recurrent, and pre‐ or post‐chemotherapy. The difference could significantly help the decision on the treatment. Moreover, in a study the NLR values on the basis of sampling time showed different results. Elevated NLR has been associated with poor prognosis in pre‐operative sampling, while post‐operative sampling showed no significant difference [65]. Fourth, most studies focused on GBM and included all three other grades in a single group. We could not determine an index for early diagnosis of early stages of glioma. Fifth, the current meta‐analysis did not obtain information on IDH1 or other glioma‐associated mutations that could alter the inflammatory status. Their relationship with the ratios still remains unknown. Sixth, due to the lack of individual data of patients we could not conclude a promising cutoff value for each inflammatory marker. Finally, aside from the heterogeneity of the included studies, potential sources of bias should be considered. Systemic effects of inflammatory response due to comorbid pathology (e.g., infection, autoimmune disease) can potentially act as confounders of leukocyte ratio patterns but were not well controlled in most included studies. Moreover, lifestyle (e.g., physical activity, smoking)and comorbidities could influence all PLR, NLR, dNLR, LMR, and MLR values, and must be better controlled in future studies.

## 5 Conclusion

This systematic review and meta‐analysis comprehensively evaluated the role of peripheral blood leukocyte ratios, including NLR, PLR, dNLR, LMR, and MLR, as potential biomarkers for brain gliomas. Our findings revealed significant increases in NLR, PLR, and dNLR indices among glioma patients compared to healthy controls. In particular, NLR demonstrated the highest diagnostic and prognostic accuracy, with specific cut‐off values and corresponding sensitivities and specificities providing a basis for clinical application. Although the LMR and MLR indices were less conclusive, our findings support the broader utility of inflammatory ratios in glioma diagnosis and prognosis. Further research is needed to refine these markers for clinical use, explore their underlying mechanisms, and validate their applicability across diverse populations and glioma subtypes.

## Author Contributions

**Fatemeh Hasani:** conceptualization (equal), data curation (equal), formal analysis (equal), investigation (equal), methodology (equal), project administration (equal), writing – original draft (equal), writing – review and editing (equal). **Kimia Jazi:** investigation (equal), methodology (equal), writing – original draft (equal). **Kimia Vakili:** formal analysis (equal), writing – review and editing (equal). **Armin Tafazolimoghadam:** investigation (equal), methodology (equal), writing – original draft (equal). **Mehrad Namazee:** investigation (equal), methodology (equal), writing – original draft (equal). **Mahdi Masrour:** conceptualization (equal), data curation (equal), formal analysis (equal), investigation (equal), methodology (equal), project administration (equal), writing – original draft (equal). **Mohammadreza Ghanbari Boroujeni:** data curation (equal), investigation (equal), methodology (equal). **Hossein Gandomkar:** data curation (equal), writing – original draft (equal). **Antonio L. Teixeira:** investigation (equal), writing – original draft (equal), writing – review and editing (equal). **Erfan Ghoodjani:** data curation (equal), writing – original draft (equal). **Mohammad Samadian:** data curation (equal), writing – review and editing (equal). **Fatemeh Sayehmiri:** methodology (equal), writing – review and editing (equal). **Seyed Ali Mousavinejad:** supervision (equal), writing – review and editing (equal).

## Funding

The authors have nothing to report.

## Ethics Statement

The authors have nothing to report. The ethical approve number for this study of Skull Base Research Center, Loghman Hakim Hospital, Shahid Beheshti University of Medical Sciences, Tehran, Iran is IR.SBMU.RETECH.REC.1403.545.

## Consent

The authors have nothing to report.

## Conflicts of Interest

The authors declare no conflicts of interest.

## Supporting information


**Table S1:** The complete search algorithms for PubMed, Web of Sciences, and Scopus library are as follows.
**Table S2:** Quality assessment of included studies.
**Table S3:** The characteristics of included studies.

## Data Availability

All data generated or analysed during this study is included in this published article and its Supporting Information files.
