# Supplementary material for: Peripheral Blood Leukocyte Ratios as Novel Biomarkers in Brain Glioma: A Comprehensive Systematic Review and Meta‐Analysis
Source: J Cell Mol Med. 2026 Jan 8;30(1):e70974. doi: 10.1111/jcmm.70974 (PMC12780876; doi:10.1111/jcmm.70974)
Supplement: Supplementary file 1 — Table S1: The complete search algorithms for PubMed, Web of Sciences, and Scopus library are as follows. Table S2: Quality assessment of included studies. Table S3: The characteristics of included studies. [file JCMM-30-e70974-s001.docx]

**Supplementary Information**

**Supplementary Table 1.** The complete search algorithms for PubMed, Web of Sciences, and Scopus library are as follows.

|  | **Query** | **Results (No.)**  **January 21, 2024** |
| --- | --- | --- |
| **PubMed** | | |
| #1 | (((((((((((monocyte* to lymphocyte* ratio[Title/Abstract]) OR (monocyte *-lymphocyte*[Title/Abstract])) OR (monocyte*-lymphocyte* ratio[Title/Abstract])) OR (monocyte* to lymphocyte*[Title/Abstract])) OR (monocyte*-to-lymphocyte* ratio[Title/Abstract])) OR (monocyte*-to lymphocyte* ratio[Title/Abstract])) OR (monocyte* to-lymphocyte* ratio[Title/Abstract])) OR (monocyte*/lymphocyte* ratio[Title/Abstract])) OR (monocyte*/lymphocyte*[Title/Abstract])) OR (mlr[Title/Abstract]))))))))))) OR (((((((((((platelet* to lymphocyte* ratio[Title/Abstract]) OR (platelet*-lymphocyte*[Title/Abstract])) OR (platelet*-lymphocyte* ratio[Title/Abstract])) OR (platelet* to lymphocyte*[Title/Abstract])) OR (platelet*-to-lymphocyte* ratio[Title/Abstract])) OR (platelet*-to lymphocyte* ratio[Title/Abstract])) OR (platelet* to-lymphocyte* ratio[Title/Abstract])) OR (platelet*/lymphocyte* ratio[Title/Abstract])) OR (platelet*/lymphocyte*[Title/Abstract])) OR (plr[Title/Abstract]))))))))))) OR (((((((((((neutrophil* to lymphocyte* ratio[Title/Abstract]) OR (neutrophil *-lymphocyte*[Title/Abstract])) OR (neutrophil*-lymphocyte* ratio[Title/Abstract])) OR (neutrophil* to lymphocyte*[Title/Abstract])) OR (neutrophil*-to-lymphocyte* ratio[Title/Abstract])) OR (neutrophil*-to lymphocyte* ratio[Title/Abstract])) OR (neutrophil* to-lymphocyte* ratio[Title/Abstract])) OR (neutrophil*/lymphocyte* ratio[Title/Abstract])) OR (neutrophil*/lymphocyte*[Title/Abstract])) OR (nlr[Title/Abstract]))))))))))) | 33,138 |
| #2 | (((((glioma*[Title/Abstract]) OR (Glial Cell Tumor*[Title/Abstract])) OR (Mixed Glioma*[Title/Abstract])) OR (Malignant Glioma*[Title/Abstract]))))) | 73,903 |
| #3 | #1 AND #2 | 121 |
| **Web of Science** | | |
| #1 | TS=(“monocyte* to lymphocyte* ratio” OR “monocyte *-lymphocyte*” OR “monocyte*-lymphocyte* ratio” OR “monocyte* to lymphocyte*” OR “monocyte*-to-lymphocyte* ratio” OR “monocyte*-to lymphocyte* ratio” OR “monocyte* to-lymphocyte* ratio” OR “monocyte*/lymphocyte* ratio” OR “monocyte*/lymphocyte*” OR “MLR” OR “platelet* to lymphocyte* ratio” OR “platelet*-lymphocyte*” OR “platelet*-lymphocyte* ratio” OR “platelet* to lymphocyte*” OR “platelet*-to-lymphocyte* ratio” OR “platelet*-to lymphocyte* ratio” OR “platelet* to-lymphocyte* ratio” OR “platelet*/lymphocyte* ratio” OR “platelet*/lymphocyte*” OR “PLR” OR “neutrophil* to lymphocyte* ratio” OR “neutrophil *-lymphocyte*” OR “neutrophil*-lymphocyte* ratio” OR “neutrophil* to lymphocyte*” OR “neutrophil*-to-lymphocyte* ratio” OR “neutrophil*-to lymphocyte* ratio” OR “neutrophil* to-lymphocyte* ratio” OR “neutrophil*/lymphocyte* ratio” OR “neutrophil*/lymphocyte*” OR “NLR”) |  |
| #2 | TS=(“glioma*” OR “Glial Cell Tumor*” OR “Mixed Glioma*” OR “Malignant Glioma*”) | 104,684 |
| #3 | #1 AND #2 | 109 |
| **SCOPUS** | | |
| #1 | TITLE-ABS-KEY(“monocyte* to lymphocyte* ratio” OR “monocyte *-lymphocyte*” OR “monocyte*-lymphocyte* ratio” OR “monocyte* to lymphocyte*” OR “monocyte*-to-lymphocyte* ratio” OR “monocyte*-to lymphocyte* ratio” OR “monocyte* to-lymphocyte* ratio” OR “monocyte*/lymphocyte* ratio” OR “monocyte*/lymphocyte*” OR “MLR” OR “platelet* to lymphocyte* ratio” OR “platelet*-lymphocyte*” OR “platelet*-lymphocyte* ratio” OR “platelet* to lymphocyte*” OR “platelet*-to-lymphocyte* ratio” OR “platelet*-to lymphocyte* ratio” OR “platelet* to-lymphocyte* ratio” OR “platelet*/lymphocyte* ratio” OR “platelet*/lymphocyte*” OR “PLR” OR “neutrophil* to lymphocyte* ratio” OR “neutrophil *-lymphocyte*” OR “neutrophil*-lymphocyte* ratio” OR “neutrophil* to lymphocyte*” OR “neutrophil*-to-lymphocyte* ratio” OR “neutrophil*-to lymphocyte* ratio” OR “neutrophil* to-lymphocyte* ratio” OR “neutrophil*/lymphocyte* ratio” OR “neutrophil*/lymphocyte*” OR “NLR”) |  |
| #2 | TITLE-ABS-KEY(“glioma*” OR “Glial Cell Tumor*” OR “Mixed Glioma*” OR “Malignant Glioma*”) | 109,092 |
| #3 | #1 AND #2 | 136 |
| **Total** | | 338 |
| **Total without duplicates** | | 183 |

**Supplementary Table 2.** Quality assessment of included studies.

| Author, year | Selection | | | | Comparability | Exposure | | | Overall score |
| --- | --- | --- | --- | --- | --- | --- | --- | --- | --- |
|  | Case definition | Representativeness | Selection of Controls | Definition of Controls |  | Ascertainment of exposure | Same method of ascertainment | Non-Response rate |  |
| M Wang, 2024 | * | * | * | * | ** | * | b | * | 8 |
| Y Yang, 2023 | * | * | * | * | ** | * | * | * | 9 |
| P Jarmuzek, 2023 | * | * | * | * | * * | * | * | * | 9 |
| F Chen, 2022 | * | * | * | * | ** | * | b | * | 8 |
| U. Yuksel, 2021 | * | * | * | * | ** | * | b | * | 8 |
| G. Xu, 2021 | * | * | * | * | ** | * | b | * | 8 |
| D. Xiao, 2021 | * | * | * | * | ** | * | b | * | 8 |
| G. Sharma, 2021 | * | * | * | * | ** | * | * | * | 9 |
| A. Kayhan, 2019 | * | * | * | * | ** | * | * | * | 9 |
| O. Baran, 2019 | * | * | * | * | ** | * | b | * | 8 |
| S. Zheng, 2017 | * | * | * | * | ** | * | b | * | 8 |
| PM Bracci, 2022 | * | * | * | * | ** | * | * | * | 9 |
| V. Subeikshanan, 2016 | * | * | * | * | * * | * | * | * | 9 |

**Supplementary Table 3.** The characteristics of included studies.

1. **PLR**

| **Author, year** | **Country** | **Case group** | **Control group** | **Case Age (mean, SD)** | **Control Age (mean, SD)** | **Case Female %** | **Control Female %** | **Case N.** | **Control N.** | **PLR mean case** | **PLR SD case** | **PLR mean Control** | **PLR SD Control** | **PLR median case** | **PLR range case** | **PLR Q1,Q3 case** | **PLR median Control** | **PLR range Control** | **PLR Q1,Q3 control** | **AUC** |
| --- | --- | --- | --- | --- | --- | --- | --- | --- | --- | --- | --- | --- | --- | --- | --- | --- | --- | --- | --- | --- |
| M Wang, 2024 | China | glioma (Gliobalstoma, Oligodendroma, Astrocytoma, Ventricular meningioma) | healthy controls | 52.9 ± 14.8 | 54.0 ± 8.98 | 40 | 62.7 | 55 | 75 |  |  |  |  | 117 | 96.73-140.95 |  | 125.63 | 103.04-153.33 |  |  |
| Y Yang, 2023 | China | glioma | Trigeminal neuralgia | 48 (8–74) | 58 (19,82) | 39.72 | 59.09% | 141 | 66 |  |  |  |  | 119.48 | 41.25-776 |  | 107.8 | 48.81-409.5 |  |  |
|  |  |  | Brain metastasis | 48 (8–74) | 58 (19,82) | 39.72 | 59.09% | 141 | 66 |  |  |  |  | 119.48 | 41.25-776 |  | 127.8 | 27.59–278.6 |  |  |
|  |  |  | meningioma | 48 (8–74) | 53 (5–81) | 39.72 | 74.12 | 141 | 313 |  |  |  |  | 119.48 | 41.25-776 |  | 110.32 | 33.33–395.24 |  |  |
|  |  |  | Craniopharyngioma | 48 (8–74) | 49 (19–66) | 39.72 | 57.14 | 141 | 14 |  |  |  |  | 119.48 | 41.25-776 |  | 75.58 | 43.64–350.8 |  |  |
|  |  |  | Ependymoma | 48 (8–74) | 47.5 (4–76) | 39.72 | 60 | 141 | 17 |  |  |  |  | 119.48 | 41.25-776 |  | 87.48 | 40.2–226 |  |  |
|  |  |  | Spinal meningioma | 48 (8–74) | 55.5 (22–83) | 39.72 | 17.65 | 141 | 19 |  |  |  |  | 119.48 | 41.25-776 |  | 134 | 63.6–204.8 |  |  |
|  |  |  | Acoustic neuroma | 48 (8–74) | 54.5 (15–83) | 39.72 | 61.29 | 141 | 93 |  |  |  |  | 119.48 | 41.25-776 |  | 110.09 | 44.71–476.32 |  |  |
| P Jarmuzek, 2023 | Poland | GBM | healthy controls | 66.0 ± 10.56 | 71.7 ± 4.9 | 60 | 55 | 50 | 40 | 173 | 120 | 123 | 46 | 151 |  |  | 112 |  |  | 0.589 |
| F Chen, 2022 | China | glioma | healthy controls | 46.4 (3-87) | 41.9 (6-85) | 41.56 | 44.51 | 1061 | 182 |  |  |  |  | 131.22 | 35.87-363.64 |  | 112.1 | 54.33-228.08 |  | 0.5213 |
|  |  |  | meningioma | 46.4 (3-87) | 51.5 (16-85) | 41.56 | 74.2 | 1061 | 1271 |  |  |  |  | 131.22 | 35.87-363.64 |  | 130.76 | 46.83-343.64 |  |  |
|  |  |  | Pituitary Adenomas | 46.4 (3-87) | 43.6 (16-77) | 41.56 | 48.1 | 1061 | 357 |  |  |  |  | 131.22 | 35.87-363.64 |  | 120.25 | 53.43262.97 |  |  |
|  |  |  | Schwannoma | 46.4 (3-87) | 48.6 (14-85) | 41.56 | 54.7 | 1061 | 316 |  |  |  |  | 131.22 | 35.87-363.64 |  | 116.25 | 54.33255.45 |  |  |
|  |  |  | TN/HS | 46.4 (3-87) | 52.9 (21-83) | 41.56 | 54.1 | 1061 | 96 |  |  |  |  | 131.22 | 35.87-363.64 |  | 111.43 | 54.32225.18 |  |  |
| U. Yuksel, 2021 | Turkey | glioblastoma | solitary brain metastasis | 59.50 (13.20) | 61.71 (9.94) | 17.2 | 6.9 | 12 | 17 | 17.96 | 15.77 | 18.04 | 14.72 |  |  |  |  |  |  |  |
| G. Xu, 2021 | China | glioma | health examination participants with matched age, sex, initial diseases | | |  |  | 45 | 45 |  |  |  |  | 139.78 |  | 96.59,205.84 | 101.79 |  | 85.64,123.25 | 0.689 |
| D. Xiao, 2021 | China | Necrotic glioblastoma | Brain abscess | 50.43 (13.31) | 44.06 (16.24) | 43 | 25 | 86 | 32 | 125.03 |  | 154.21 |  |  |  | 94.27,175.27 |  |  | 114.01,248.80 |  |
| G. Sharma, 2021 | India | glioma | healthy controls | 42 (3–87) | 41 (7–82) | 38.32 | 44.16 | 154 | 107 |  |  |  |  | 120.66 | 32.79-510.26 |  | 111.75 | 25.6-325.6 |  | 0.460037 |
|  |  |  | Acoustic neuroma | 42 (3–87) | 49.5 (22–78) | 38.32 | 61 | 154 | 36 |  |  |  |  | 120.66 | 32.79-510.26 |  | 111.39 | 117.14–119.46 |  |  |
|  |  |  | Meningioma | 42 (3–87) | 54.5 (19–86) | 38.32 | 60.3 | 154 | 58 |  |  |  |  | 120.66 | 32.79-510.26 |  | 130.8 | 51.53–615.18 |  |  |
| A. Kayhan, 2019 | Turkey | GBM | control | 56.16 (16.45) | 32 (10.8) | 43.2 | 46.7 | 37 | 30 | 151.3 | 61.6 | 119 | 35.3 |  |  |  |  |  |  |  |
|  |  |  | intracranial supratentorial metastasis | 56.16 (16.45) | 56.09 (9.41) | 43.2 | 34.4 | 37 | 32 | 151.3 | 61.6 | 203.2 | 105.5 |  |  |  |  |  |  | 0.66 |
|  |  |  | Temporal lobe epilepsy | 56.16 (16.45) | 25.53 ± 10.99 | 43.2 | 43.6 | 37 | 39 | 151.3 | 61.6 | 110.7 | 41.7 |  |  |  |  |  |  |  |
|  |  |  | Meningioma | 56.16 (16.45) | 57.50 ± 12.39 | 43.2 | 59.4 | 37 | 32 | 151.3 | 61.6 | 150.2 | 71.4 |  |  |  |  |  |  |  |
| O. Baran, 2019 | Turkey | patients who underwent resective surgery for GBM | metastasis | 51.65 (16.71) | 57.30 (10.20) | 40 | 30 | 80 | 70 | 156.2 | 69.14 | 203.78 | 125.29 |  |  |  |  |  |  | 0.61 |
| S. Zheng, 2017 | China | Glioma | Healthy Controls | 43 (1–85) median range | 42 (6–84) median range | 44.53 | 44.28 | 750 | 682 |  |  |  |  | 123.92 | 19.67–482.46 |  | 108.6 | 46.10–241.27 |  |  |

1. **NLR**

| **Author, year** | **Country** | **Case group** | **Control group** | **Case Age (mean, SD)** | **Control Age (mean, SD)** | **Case Female %** | **Control Female %** | **Case N.** | **Control N.** | **NLR mean case** | **NLR SD case** | **NLR mean Control** | **NLR SD Control** | **NLR median case** | **NLR range case** | **NLR Q1,Q3 case** | **NLR IQR case** | **NLR median Control** | **NLR range Control** | **NLR Q1,Q3 control** | **NLR IQR control** | **NLR (HR, RR , ..)** | **Upper Ci** | **lower CI** | **P-value** | **AUC** |
| --- | --- | --- | --- | --- | --- | --- | --- | --- | --- | --- | --- | --- | --- | --- | --- | --- | --- | --- | --- | --- | --- | --- | --- | --- | --- | --- |
| M Wang, 2024 | China | glioma (Gliobalstoma, Oligodendroma, Astrocytoma, Ventricular meningioma) | healthy controls | 52.9 ± 14.8 | 54.0 ± 8.98 | 40 | 62.7 | 55 | 75 |  |  |  |  | 1.82 | 1.51-2.42 |  |  | 1.67 | 1.34-2.00 |  |  |  |  |  | <0.05 |  |
| Y Yang, 2023 | China | glioma | Trigeminal neuralgia | 48 (8–74) | 58 (19-82) | 39.72 | 59.09 | 141 | 66 |  |  |  |  | 2.81 | 0.97-28.74 |  |  | 2.11 | 0.97-18.39 |  |  |  |  |  | <0.05 |  |
|  |  |  | meningioma | 48 (8–74) | 53 (5–81) | 39.72 | 74.12 | 141 | 313 |  |  |  |  | 2.81 | 0.97-28.74 |  |  | 2.13 | 0.6–18.89 |  |  |  |  |  | <0.05 | 0.6505 |
|  |  |  | Brain metastasis | 48 (8–74) | 59 (39–78) | 39.72 | 51.28 | 141 | 39 |  |  |  |  | 2.81 | 0.97-28.74 |  |  | 3.65 | 1.2-16.38 |  |  |  |  |  | <0.05 |  |
|  |  |  | Craniopharyngioma | 48 (8–74) | 49 (19–66) | 39.72 | 57.14 | 141 | 14 |  |  |  |  | 2.81 | 0.97-28.74 |  |  | 1.74 | 0.9–15.95 |  |  |  |  |  | <0.05 |  |
|  |  |  | Ependymoma | 48 (8–74) | 47.5 (4–76) | 39.72 | 60 | 141 | 17 |  |  |  |  | 2.81 | 0.97-28.74 |  |  | 2.22 | 0.93–8.92 |  |  |  |  |  | <0.05 |  |
|  |  |  | Spinal meningioma | 48 (8–74) | 55.5 (22–83) | 39.72 | 17.65 | 141 | 19 |  |  |  |  | 2.81 | 0.97-28.74 |  |  | 2.13 | 0.84–4.29 |  |  |  |  |  | <0.05 |  |
|  |  |  | Acoustic neuroma | 48 (8–74) | 54.5 (15–83) | 39.72 | 61.29 | 141 | 93 |  |  |  |  | 2.81 | 0.97-28.74 |  |  | 2.11 | 0.83–4.79 |  |  |  |  |  | <0.05 |  |
| P Jarmuzek, 2023 | Poland | GBM | healthy controls | 66.0 ± 10.56 | 71.7 ± 4.9 | 60 | 55 | 50 | 40 | 8.08 | 7.26 | 1.98 | 1.53 | 5.53 |  |  |  | 1.74 |  |  |  | 7.875 (RR) | 20.148 | 3.333 | <0.001 | 0.831 |
| F Chen, 2022 | China | glioma | Healthy controls | 46.4 (3-87) | 41.9 (6-85) | 41.56 | 44.51 | 1061 | 182 |  |  |  |  | 2.29 | 0.58-11.14 |  |  | 1.49 | 0.71-2.88 |  |  |  |  |  | <0.05 | 0.8099 |
|  |  | GBM | Healthy controls | 46.4 (3-87) |  |  |  |  |  |  |  |  |  |  |  |  |  |  |  |  |  |  |  |  |  | 0.9585 |
|  |  | glioma | Pituitary Adenomas | 46.4 (3-87) | 43.6 (16-77) | 41.56 | 48.1 | 1061 | 357 |  |  |  |  | 2.29 | 0.58-11.14 |  |  | 1.53 | 0.63-2.79 |  |  |  |  |  |  |  |
|  |  | glioma | TN/HS | 46.4 (3-87) | 52.9 (21-83) | 41.56 | 54.1 | 1061 | 96 |  |  |  |  | 2.29 | 0.58-11.14 |  |  | 1.49 | 0.72-2.32 |  |  |  |  |  |  |  |
|  |  | glioma | Schwannoma | 46.4 (3-87) | 48.6 (14-85) | 41.56 | 74.2 | 1061 | 316 |  |  |  |  | 2.29 | 0.58-11.14 |  |  | 1.51 | 0.64-2.72 |  |  |  |  |  |  |  |
|  |  | glioma | meningioma | 46.4 (3-87) | 51.5 (16-85) | 41.56 | 74 | 1061 | 1271 |  |  |  |  | 2.29 | 0.58-11.14 |  |  | 1.83 | 0.77-7.47 |  |  |  |  |  |  |  |
| PM Bracci, 2022 | USA | Immune Profle Study (IPS) glioma patients | Adult Glioma Study (AGS) controls | 50.54 (15.40) | 51.77 (15.53) | 38.8 | 45.6 | 139 | 454 | 4.97 | 5.2 | 1.84 | 1.14 | 2.69 |  | 1.65,6.14 | 4.49 | 1.57 |  | 1.16,2.21 | 1.05 |  |  |  | <0.001 |  |
| U. Yuksel, 2021 | Turkey | glioblastoma | solitary brain metastasis | 59.50 (13.20) | 61.71 (9.94) | 17.2 | 6.9 | 12 | 17 |  |  |  |  | 4.07 | 1.47-31.75 |  |  | 4.19 | 1.65-12.82 |  |  |  |  |  | 0.506 |  |
| G. Xu, 2021 | China | patients with glioma who underwent neurosurgical resection | health examination participants with matched age, sex, initial diseases | | | |  | 45 | 45 |  |  |  |  | 3.49 |  | 2.19,4.82 |  | 1.54 |  | 1.13,1.95 |  |  |  |  | <.001 | 0.889 |
| D. Xiao, 2021 | China | Necrotic glioblastoma | Brain abscess | 50.43 (13.31) | 44.06 (16.24) | 43 | 25 | 86 | 32 | 2.81 |  | 5.35 |  |  |  | 1.95,6.23 |  |  |  | 2.16,11.56 |  |  |  |  | 0.048 |  |
| G. Sharma, 2021 | India | glioma | healthy controls | 42 (3–87) | 41 (7–82) | 38.32 | 44.16 | 154 | 107 |  |  |  |  | 2.47 | 0.44–15.04 |  |  | 1.52 | 0.42-4.36 |  |  |  |  |  | <0.05 | 0.656604 |
|  |  |  | Meningioma | 42 (3–87) | 54.5 (19–86) | 38.32 | 60 | 154 | 58 |  |  |  |  | 2.47 | 0.44–15.04 |  |  | 1.94 | 0.84–4.66 |  |  |  |  |  |  |  |
|  |  |  | Acoustic neuroma | 42 (3–87) | 49.5 (22–78) | 38.32 | 60 | 154 | 36 |  |  |  |  | 2.47 | 0.44–15.04 |  |  | 1.94 | 1.75–1.9 |  |  |  |  |  |  |  |
| A. Kayhan, 2019 | Turkey | GBM | Healthy Control | 56.16 (16.45) | 32 (10.8) | 43.2 | 46.7 | 37 | 30 | 4.12 | 2.5 | 1.81 | 0.5 |  |  |  |  |  |  |  |  |  |  |  | <0.05 |  |
|  |  |  | intracranial supratentorial metastasis | 56.16 (16.45) | 56.09 (9.41) | 43.2 | 34.4 | 37 | 32 | 4.12 | 2.5 | 5.88 | 4.8 |  |  |  |  |  |  |  |  |  |  |  |  | 0.59 |
|  |  |  | Temporal lobe epilepsy | 56.16 (16.45) | 25.53 ± 10.99 | 43.2 | 43.6 | 37 | 39 | 4.12 | 2.5 | 1.92 | 1.1 |  |  |  |  |  |  |  |  |  |  |  |  |  |
|  |  |  | Meningioma | 56.16 (16.45) | 57.50 ± 12.39 | 43.2 | 59.4 | 37 | 32 | 4.12 | 2.5 | 4.81 | 4.4 |  |  |  |  |  |  |  |  |  |  |  |  |  |
| O. Baran, 2019 | Turkey | GBM | metastasis | 51.65 (16.71) | 57.30 (10.20) | 40 | 30 | 80 | 70 | 6.91 | 3.14 | 7.03 | 3.15 |  |  |  |  |  |  |  |  |  |  |  | 0.05 | 0.58 |
| S. Zheng, 2017 | China | Glioma | Healthy Controls | 43 (1–85) median range | 42 (6–84) median range | 44.53 | 44.28 | 750 | 682 |  |  |  |  | 2.25 | 0.19–22.47 |  |  | 1.49 | 0.73–3.00 |  |  |  |  |  | <0.05 |  |
| V. Subeikshanan, 2016 | India | GBM | Healthy Controls | _ | 25.95 (±7.5) |  | 17.13 | 36 | 216 | 2.67 | 2.2 | 1.97 | 0.84 |  |  |  |  |  |  |  |  |  |  |  | 0.028 |  |
|  |  | supratentorial gliomas | Healthy Controls | 32.89(±16.3) | 25.95 (±7.5) | male to female distribution was 1.33: 1 | 17.13 | 70 | 216 | 2.44 | 1.92 | 1.97 | 0.84 |  |  |  |  |  |  |  |  |  |  |  | 0.051 |  |

1. **LMR**

| **Authoe, year** | **Country** | **Case group** | **Control group** | **Case Age (mean, SD)** | **Control Age (mean, SD)** | **Case Female %** | **Control Female %** | **Case N.** | **Control N.** | **LMR mean case** | **LMR SD case** | **LMR mean Control** | **LMR SD Control** | **LMR median case** | **LMR range case** | **LMR Q1,Q3 case** | **LMR IQR case** | **LMR median Control** | **LMR range Control** | **LMR Q1,Q3 control** | **LMR IQR control** | **LMR (HR, RR , ..)** | **Upper Ci** | **lower CI** | **P-value** | **AUC** |
| --- | --- | --- | --- | --- | --- | --- | --- | --- | --- | --- | --- | --- | --- | --- | --- | --- | --- | --- | --- | --- | --- | --- | --- | --- | --- | --- |
| Y Yang, 2023 | China | glioma | Trigeminal neuralgia | 48 (8–74) | 58 (19,82) | 39.72 | 59.09% | 141 | 66 |  |  |  |  | 4.02 | 0.92–16.33 |  |  | 4.42 | 0.96–12.5 |  |  |  |  |  | < 0.05 |  |
|  |  |  | Brain metastasis | 48 (8–74) | 58 (19,82) | 39.72 | 59.09% | 141 | 66 |  |  |  |  | 4.02 | 0.92–16.33 |  |  | 3.87 | 0.38–12.56 |  |  |  |  |  | < 0.05 |  |
|  |  |  | meningioma | 48 (8–74) | 53 (5–81) | 39.72 | 74.12 | 141 | 313 |  |  |  |  | 4.02 | 0.92–16.33 |  |  | 4.91 | 0.92–12.06 |  |  |  |  |  | < 0.05 |  |
|  |  |  | Craniopharyngioma | 48 (8–74) | 49 (19–66) | 39.72 | 57.14 | 141 | 14 |  |  |  |  | 4.02 | 0.92–16.33 |  |  | 6.4 | 1.05–8.59 |  |  |  |  |  | < 0.05 |  |
|  |  |  | Ependymoma | 48 (8–74) | 47.5 (4–76) | 39.72 | 60 | 141 | 17 |  |  |  |  | 4.02 | 0.92–16.33 |  |  | 4.75 | 2.64–10.45 |  |  |  |  |  | < 0.05 |  |
|  |  |  | Spinal meningioma | 48 (8–74) | 55.5 (22–83) | 39.72 | 17.65 | 141 | 19 |  |  |  |  | 4.02 | 0.92–16.33 |  |  | 5.64 | 2.24–7.81 |  |  |  |  |  | < 0.05 |  |
|  |  |  | Acoustic neuroma | 48 (8–74) | 54.5 (15–83) | 39.72 | 61.29 | 141 | 93 |  |  |  |  | 4.02 | 0.92–16.33 |  |  | 4.71 | 1.72–9.83 |  |  |  |  |  | < 0.05 |  |
| P Jarmuzek, 2023 | Poland | GBM | healthy controls | 66.0 ± 10.56 | 71.7 ± 4.9 | 60 | 55 | 50 | 40 | 4.21 | 5.9 | 3.97 | 1.27 | 2.82 |  |  |  | 3.73 |  |  |  | 0.438 (RR) | 0.438 | 0.252 | 0.725 | 0.69 |
| F Chen, 2022 | China | glioma | Healthy controls | 46.4 (3-87) | 41.9 (6-85) | 41.56 | 44.51 | 1061 | 182 |  |  |  |  | 5.28 | 1.04-11.90 |  |  | 6.83 | 1.95-13.86 |  |  |  |  |  | <0.05 | 0.7045 |
|  |  |  | Pituitary Adenomas | 46.4 (3-87) | 43.6 (16-77) | 41.56 | 48.1 | 1061 | 357 |  |  |  |  | 5.28 | 1.04-11.90 |  |  | 6.62 | 1.73-14.14 |  |  |  |  |  | <0.05 |  |
|  |  |  | TN/HS | 46.4 (3-87) | 52.9 (21-83) | 41.56 | 54.1 | 1061 | 96 |  |  |  |  | 5.28 | 1.04-11.90 |  |  | 6.99 | 1.85-13.79 |  |  |  |  |  | <0.05 |  |
|  |  |  | Schwannoma | 46.4 (3-87) | 48.6 (14-85) | 41.56 | 74.2 | 1061 | 316 |  |  |  |  | 5.28 | 1.04-11.90 |  |  | 6.62 | 1.75-13.63 |  |  |  |  |  | <0.05 |  |
|  |  |  | meningioma | 46.4 (3-87) | 51.5 (16-85) | 41.56 | 74 | 1061 | 1271 |  |  |  |  | 5.28 | 1.04-11.90 |  |  | 5.88 | 1.30-13.23 |  |  |  |  |  | <0.05 |  |
| PM Bracci, 2022 | USA | Immune Profle Study (IPS) glioma patients | Adult Glioma Study (AGS) controls | 50.54 (15.40) | 51.77 (15.53) | 38.8 | 45.6 | 139 | 454 | 4.97 | 5.2 | 1.84 | 1.14 | 2.69 |  | 1.65,6.14 | 2.96 | 1.57 |  | 1.16,2.21 | 2.61 |  |  |  | <0.001 |  |
| D. Xiao, 2021 | China | Necrotic glioblastoma | Brain abscess | 50.43 (13.31) | 44.06 (16.24) | 43 | 25 | 86 | 32 | 0.4 | 0.39 | 0.51 | 0.36 |  |  |  |  |  |  |  |  |  |  |  | 0.172 |  |
| G. Sharma, 2021 | India | glioma | healthy controls | 42 (3–87) | 41 (7–82) | 38.32 | 44.16 | 154 | 107 |  |  |  |  | 4.34 | 0.3-18.9 |  |  | 5.6 | 0.45–13.4 |  |  |  |  |  | <0.05 | 0.411474 |
|  |  |  | Meningioma | 42 (3–87) | 54.5 (19–86) | 38.32 | 60 | 154 | 58 |  |  |  |  | 4.34 | 0.3-18.9 |  |  | 4.92 | 1.68-10.38 |  |  |  |  |  |  |  |
|  |  |  | Acoustic neuroma | 42 (3–87) | 49.5 (22–78) | 38.32 | 61 | 154 | 36 |  |  |  |  | 4.34 | 0.3-18.9 |  |  | 5.56 | 7–22.01 |  |  |  |  |  |  |  |
| O. Baran, 2019 | Turkey | patients who underwent resective surgery for GBM | metastasis | 51.65 (16.71) | 57.30 (10.20) | 40 | 30 | 80 | 70 | 3.56 | 1.89 | 2.8 | 1.68 |  |  |  |  |  |  |  |  |  |  |  | 0.01 | 0.64 |
| S. Zheng, 2017 | China | Glioma | Healthy Controls | 43 (1–85) median range | 42 (6–84) median range | 44.53 | 44.28 | 750 | 682 |  |  |  |  | 4.09 | 0.52–16.43 |  |  | 6.18 | 3.51–13.16 |  |  |  |  |  | <0.05 |  |

1. **dNLR**

| **Author, year** | **Country** | **Case group** | **Control group** | **Case Age (mean, SD)** | **Control Age (mean, SD)** | **Case Female %** | **Control Female %** | **Case N.** | **Control N.** | **dNLR mean case** | **dNLR SD case** | **dNLR mean Control** | **dNLR SD Control** | **dNLR median case** | **dNLR range case** | **dNLR Q1,Q3 case** | **dNLR median Control** | **dNLR range Control** | **dNLR Q1,Q3 control** | **dNLR (HR, RR , ..)** | **Upper CI** | **lower CI** | **P-value** | **AUC** |
| --- | --- | --- | --- | --- | --- | --- | --- | --- | --- | --- | --- | --- | --- | --- | --- | --- | --- | --- | --- | --- | --- | --- | --- | --- |
| Y Yang, 2023 | China | glioma | Trigeminal neuralgia | 48 (8–74) | 58 (19,82) | 39.72 | 59.09% | 141 | 66 |  |  |  |  | 1.33 | 1.1-2.13 |  | 1.32 | 1.11-2.32 |  |  |  |  | ns |  |
|  |  |  | Brain metastasis | 48 (8–74) | 58 (19,82) | 39.72 | 59.09% | 141 | 66 |  |  |  |  | 1.33 | 1.1-2.13 |  | 1.34 | 1.09-1.97 |  |  |  |  | ns |  |
|  |  |  | meningioma | 48 (8–74) | 53 (5–81) | 39.72 | 74.12 | 141 | 313 |  |  |  |  | 1.33 | 1.1-2.13 |  | 1.29 | 1.1–2.32 |  |  |  |  |  | 0.5709 (0.5109–0.6308) |
|  |  |  | Craniopharyngioma | 48 (8–74) | 49 (19–66) | 39.72 | 57.14 | 141 | 14 |  |  |  |  | 1.33 | 1.1-2.13 |  | 1.28 | 1.16–2.03 |  |  |  |  |  |  |
|  |  |  | Ependymoma | 48 (8–74) | 47.5 (4–76) | 39.72 | 60 | 141 | 17 |  |  |  |  | 1.33 | 1.1-2.13 |  | 1.29 | 1.11–1.58 |  |  |  |  |  |  |
|  |  |  | Spinal meningioma | 48 (8–74) | 55.5 (22–83) | 39.72 | 17.65 | 141 | 19 |  |  |  |  | 1.33 | 1.1-2.13 |  | 1.27 | 1.21–1.75 |  |  |  |  |  |  |
|  |  |  | Acoustic neuroma | 48 (8–74) | 54.5 (15–83) | 39.72 | 61.29 | 141 | 93 |  |  |  |  | 1.33 | 1.1-2.13 |  | 1.3 | 1.14–1.72 |  |  |  |  |  |  |
| F Chen, 2022 | China | Glioma | HC | 46.4 (3-87) | 41.9 (6-85) | 41.56 | 44.51 | 1061 | 182 |  |  |  |  | 1.54 | 0.15-6.64 |  | 1.34 | 0.52-2.57 |  |  |  |  | <0.05 | 0.6914 (0.6491-0.7337) |
| F Chen, 2022 | China | glioma | Pituitary Adenomas | 46.4 (3-87) | 43.6 (16-77) | 41.56 | 48.1 | 1061 | 357 |  |  |  |  | 1.54 | 0.15-6.64 |  | 1.33 | 0.27-3.44 |  |  |  |  |  |  |
|  |  |  | TN/HS | 46.4 (3-87) | 52.9 (21-83) | 41.56 | 54.1 | 1061 | 96 |  |  |  |  | 1.54 | 0.15-6.64 |  | 1.35 | 0.52-4.77 |  |  |  |  |  |  |
|  |  |  | Schwannoma | 46.4 (3-87) | 48.6 (14-85) | 41.56 | 74.2 | 1061 | 316 |  |  |  |  | 1.54 | 0.15-6.64 |  | 1.25 | 0.28-3.06 |  |  |  |  |  |  |
|  |  |  | meningioma | 46.4 (3-87) | 51.5 (16-85) | 41.56 | 74 | 1061 | 1271 |  |  |  |  | 1.54 | 0.15-6.64 |  | 1.32 | 0.36-6.64 |  |  |  |  |  |  |
| D. Xiao, 2021 | China | Necrotic glioblastoma | Brain abscess | 50.43 (13.31) | 44.06 (16.24) | 43 | 25 | 86 | 32 | 1.55 |  | 1.23 |  |  |  | 1.19,2.05 |  |  | 1.10,1.88 |  |  |  | 0.051 |  |
| G. Sharma, 2021 | India | glioma | healthy controls | 42 (3–87) | 41 (7–82) | 38.32 | 44.16 | 154 | 107 |  |  |  |  | 1.61 | 0.44-15.04 |  | 1.06 | 0.32-4.36 |  |  |  |  | <0.05 | 0.656639 |
|  |  |  | Meningioma | 42 (3–87) | 54.5 (19–86) | 38.32 | 60 | 154 | 58 |  |  |  |  | 1.61 | 0.44-15.04 |  | 1.43 | 0.54–2.87 |  |  |  |  | <0.05 |  |
|  |  |  | Acoustic neuroma | 42 (3–87) | 49.5 (22–78) | 38.32 | 60 | 154 | 36 |  |  |  |  | 1.61 | 0.44-15.04 |  | 1.16 | 1.14–1.5 |  |  |  |  | <0.05 |  |
| S. Zheng, 2017 | China | Glioma | Healthy Controls | 43 (1–85) median range | 42 (6–84) median range | 44.53 | 44.28 | 750 | 682 |  |  |  |  | 1.65 | 0.03–10.73 |  | 1.21 | 0.60–2.42 |  |  |  |  | <0.05 |  |

1. **MLR**

| **Author, year** | **Country** | **Case group** | **Control group** | **Case Age (mean, SD)** | **Control Age (mean, SD)** | **Case Female %** | **Control Female %** | **Case N.** | **Control N.** | **MLR mean case** | **MLR SD case** | **MLR mean Control** | **MLR SD Control** | **MLR median case** | **MLR range case** | **MLR median Control** | **MLR range Control** |
| --- | --- | --- | --- | --- | --- | --- | --- | --- | --- | --- | --- | --- | --- | --- | --- | --- | --- |
| M Wang, 2024 | China | glioma (Gliobalstoma, Oligodendroma, Astrocytoma, Ventricular meningioma) | healthy controls | 52.9 ± 14.8 | 54.0 ± 8.98 | 40 | 62.7 | 55 | 75 |  |  |  |  | 0.27 | 0.19-0.35 | 0.17 | 0.13-0.20 |
| V. Subeikshanan, 2016 | India | GBM | Healthy Controls |  | 25.95 (±7.5) |  | 17.13 | 36 | 216 | 0.02 | 0.04 | 0.18 | 0.08 |  |  |  |  |
|  |  | supratentorial gliomas | Healthy Controls | 32.89(±16.3) | 25.95 (±7.5) | 42.86 | 17.13 | 70 | 216 | 0.18 | 0.08 | 0.03 | 0.06 |  |  |  |  |
